# Polymer Cold-Flow Improvers for Biodiesel

**DOI:** 10.3390/polym13101580

**Published:** 2021-05-14

**Authors:** Ilya Nifant’ev, Pavel Ivchenko

**Affiliations:** 1 A.V. Topchiev Institute of Petrochemical Synthesis RAS, 29 Leninsky Pr., 119991 Moscow, Russia; phpasha1@yandex.ru or; 2 Chemistry Department, M.V. Lomonosov Moscow State University, 1 Leninskie Gory Str., Building 3, 119991 Moscow, Russia

**Keywords:** biodiesel, cloud point, cold-flow property, depressants, dispersants, esters, fatty acids, pour point

## Abstract

In recent decades, biodiesel has been explored as a prospective comparable fuel to petroleum diesel for compression ignition engines. However, several drawbacks have limited the wide application of biodiesel as motor fuel, and the poor cold-flow property is one of the major problems. This problem is compounded by the diversity of the biodiesel characteristics arising from a variety of chemical compositions of biodiesel from different sources. Among the methods investigated to improve the cold-flow properties of biodiesel, the use of additives seems highly promising. Despite the significant number of publications, the potential of this method is still far from having been completely discovered or exploited. In the present review, we briefly describe the sources, chemical composition, and physico-chemical characteristics of the main types of biodiesel. Next, we discuss the examples of the use of different polymer additives for the improvement of the cold-flow characteristics of biodiesel and biodiesel/petroleum diesel blends. Additionally, we tried to assess the prospects of the polymer additives to enhance biodiesel performance. The main conclusion of this survey is that innovative and high-efficiency cold-flow improvers for biodiesel should be further developed.

## 1. Introduction

**Figure 1:**
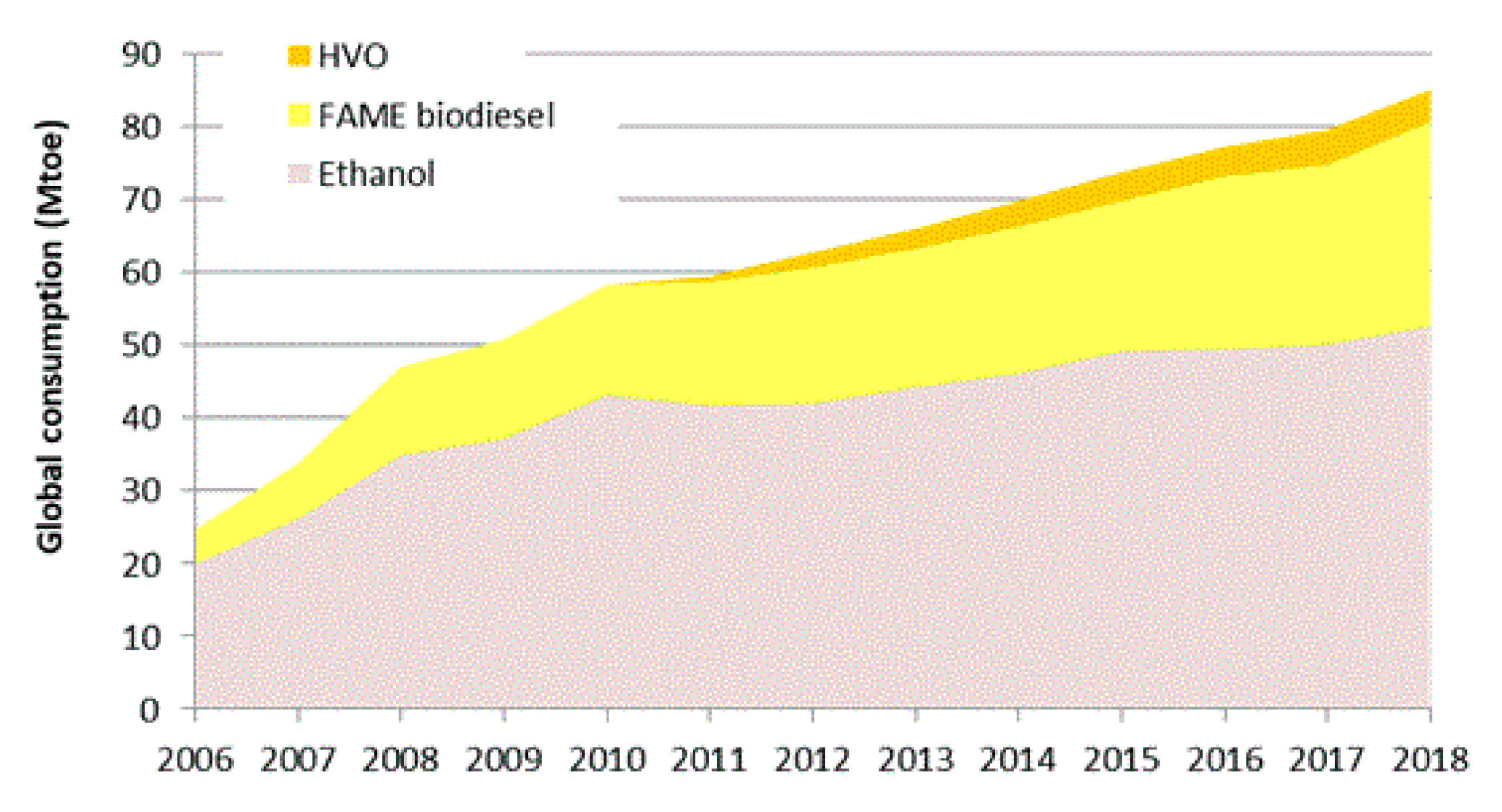
Evolution of global biofuel consumption in the road transport sector (FAME—Fatty Acid Methyl Esters, biodiesel; HVO—Hydrotreated Vegetable Oils) [3].

The concept of progressive and sustainable development stimulated the search for strategies for producing fuels, lubricants and chemicals from renewable sources. Biofuels, derived from plant biomass, are the only sustainable source of liquid fuels [1,2]. The global biofuel consumption in the road transport sector demonstrates stable growth (Figure 1) [3]. It is expected that the share of biofuels will amount to ~5% of liquid fuels for the transport industry by 2040 [4].

**Figure 2:**
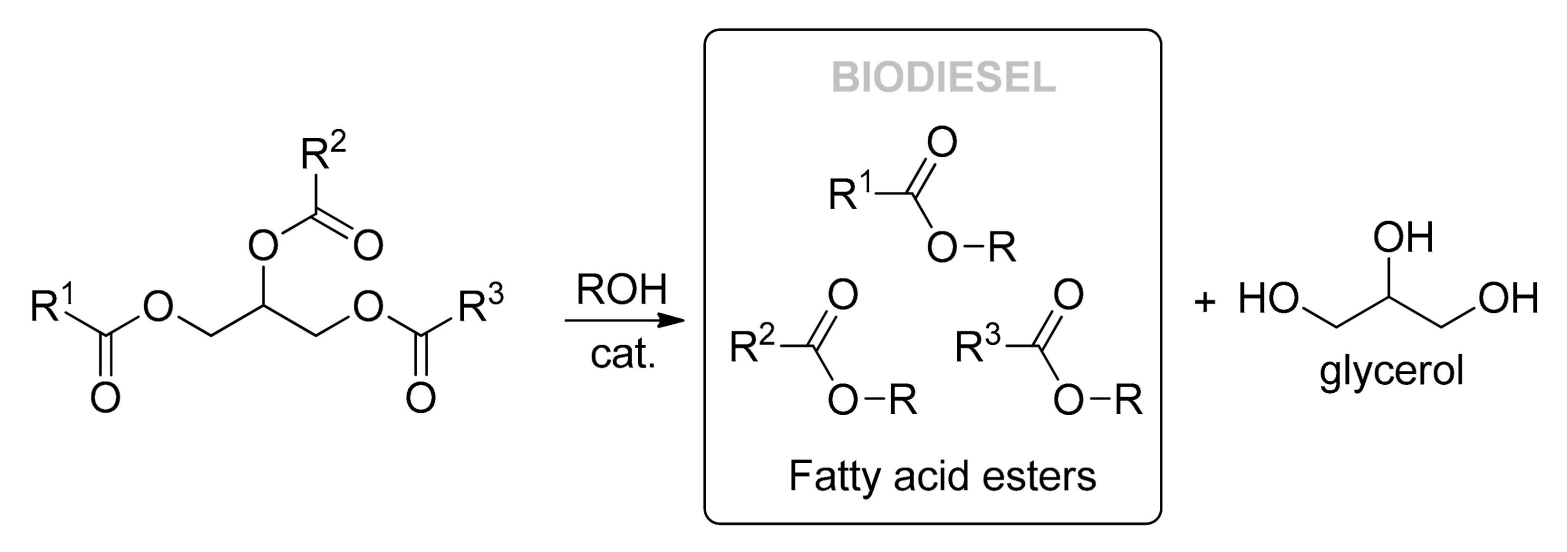
The synthesis of BD by transesterification of glycerides (oils and fats).

Biodiesel (BD) is a mixture of fatty acid (FA) alkyl esters (mainly methyl esters) obtained from oils and fats by transesterification (Figure 2). The worldwide biodiesel production in 2019 was 48 billion L [5]. The use of BD as fuel presents some advantages such as reduced particulate matter, unburnt hydrocarbon, carbon monoxide emissions, higher cetane number and better lubricity in comparison with petroleum diesel. However, the use of biodiesel is affected by its poor cold-flow properties (CFP) [6]. The impacts of cold temperatures on biodiesel in fuel systems are generally limited to start-up and operability problems that may arise when the engine and fuel system are shut down overnight. These impacts would then result from gelling or solidification in the fuel that can restrict or plug the flow of fuel in the fuel system [7].

**Figure 3:**
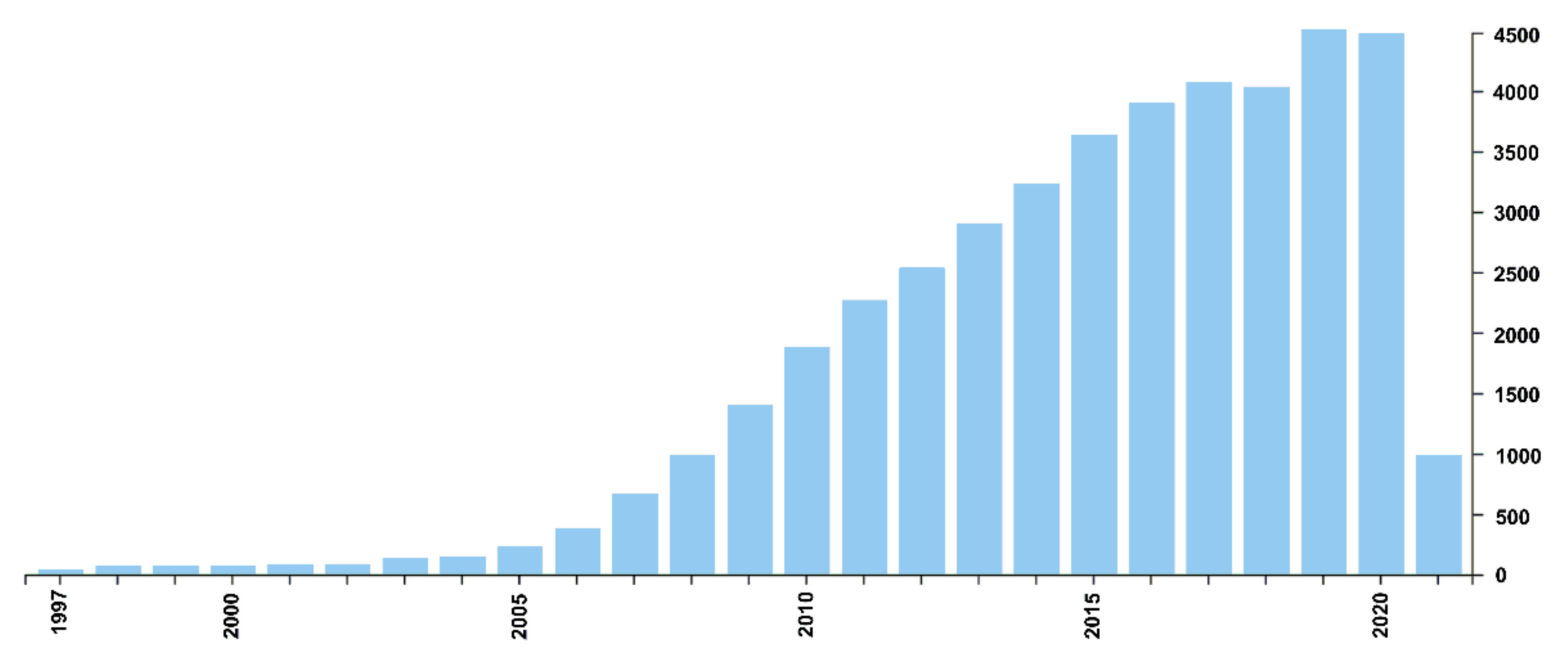
Annual numbers of scientific articles on the topic of “biodiesel.” Web of Science data (Thomson-Reuters, 15 April 2021).

The studies of BD are still extremely significant (Figure 3). Many reviews focus on the synthesis, purification, upgrading, properties and applications of BD [8,9,10,11,12,13,14,15,16,17,18,19,20,21,22,23,24,25,26,27,28,29,30,31]. The problem of improving the CFP of BD was also discussed in a number of reviews [6,32,33,34,35,36,37,38,39,40,41,42,43].

There are several ways in which the problem of poor CFP of BD can be solved. CFP of BD can be improved significantly using pre-treatment of the BD (“winterization”) [32], by mixing with petroleum diesel or blending with fatty acid esters with good CFP, and by using of the additives, cold-flow improvers (CFIs) [6,33,35,37,38,39,40,41,42,43].

Low molecular weight (MW) CFIs have been well-represented in the literature, and relatively high efficiency was demonstrated by ethanol as diluent [44], hydroxyacetone, solketal, glycerol formal [45], ozonized vegetable oil [46], triacetin [47,48], dimethyl azelate [48], branched esters of FA [49,50], monoglycerides as emulsifiers [51], mannosylerythritol lipid-A [52], branched diesters [53,54] and other compounds [6,32,33,34,35,36,37,38,39,40,41,42,43]. However, it is difficult to find a balance between the effect of the additives and the result of blending: actually, appreciable percentages of low MW additives were acting as a component of binary/ternary mixtures, which naturally have lower crystallization points [44,55,56].

On the basis of polymer science, high-MW CFIs are of greater interest to developing BD-based fuel recipes suitable for use in countries with colder climates. This approach had been effective in improving the CFP of petroleum diesel and oil products [57,58,59]. In the present review, we describe the key characteristics of the BD based on compositions of the raw materials, discuss the CFP of BD from different sources, and analyze the data on the use of polymers to improve the CFP of BD and BD blends.

## 2. Sources, Synthesis and Chemical Composition of BD

### 2.1. Oils, as Raw Material for the Production of BD

**Figure 4:**
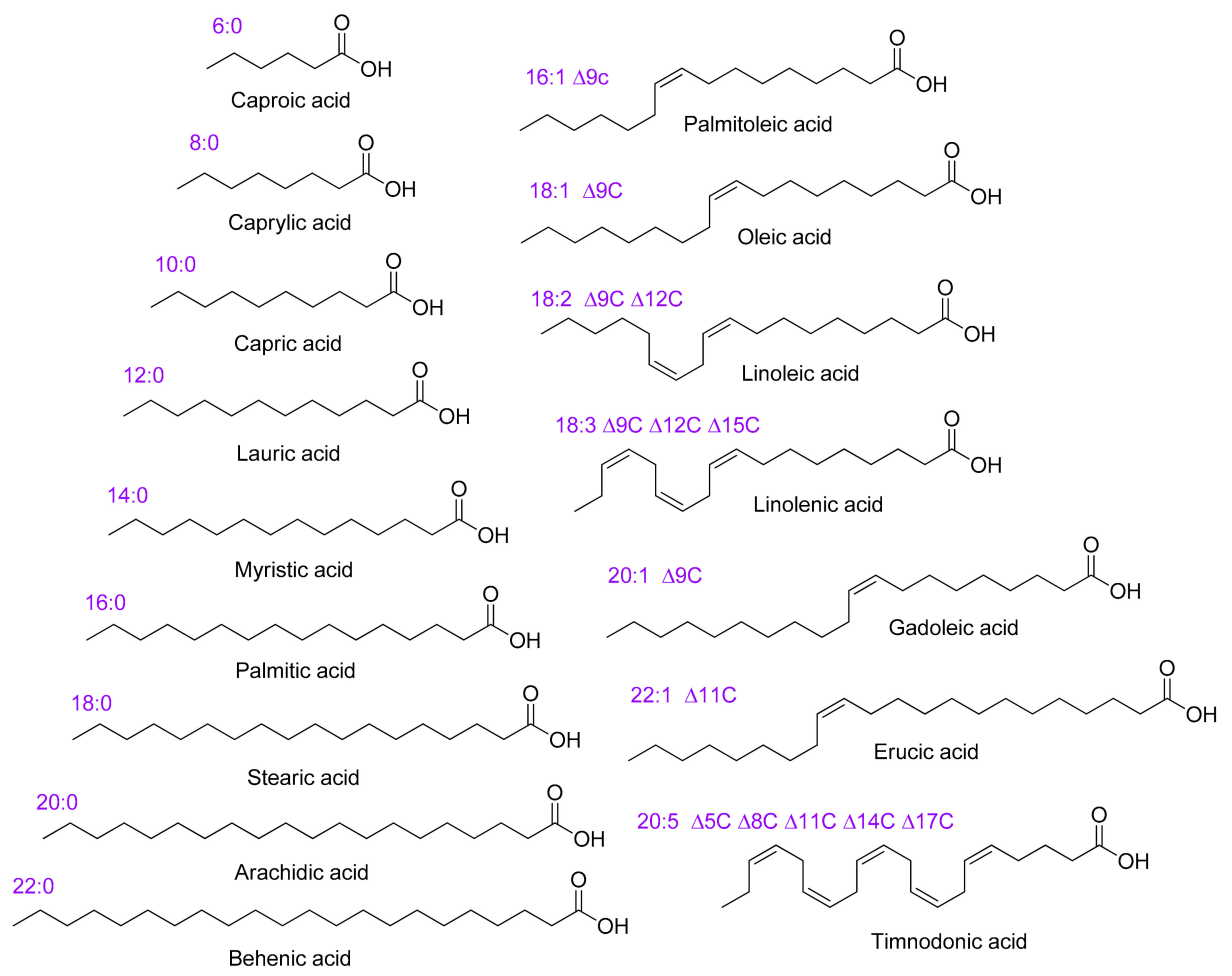
Molecular formula and lipid codes of the main fatty acids (FAs) in oils and fats used in the synthesis of BD.

BD is produced using oil from various sources, including edible vegetable oils, non-edible oils, waste oils and fats. Since the sources of the feedstocks for BD production are based on regional availabilities, the compositions of the fatty acid alkyl esters also vary due to the difference in the compositions of the saturated and unsaturated fatty acids (Figure 4) in corresponding triglycerides. In Table 1, we presented the data on the composition of glycerides that form oils used as BD feedstocks [16,60].

**Figure 5:**
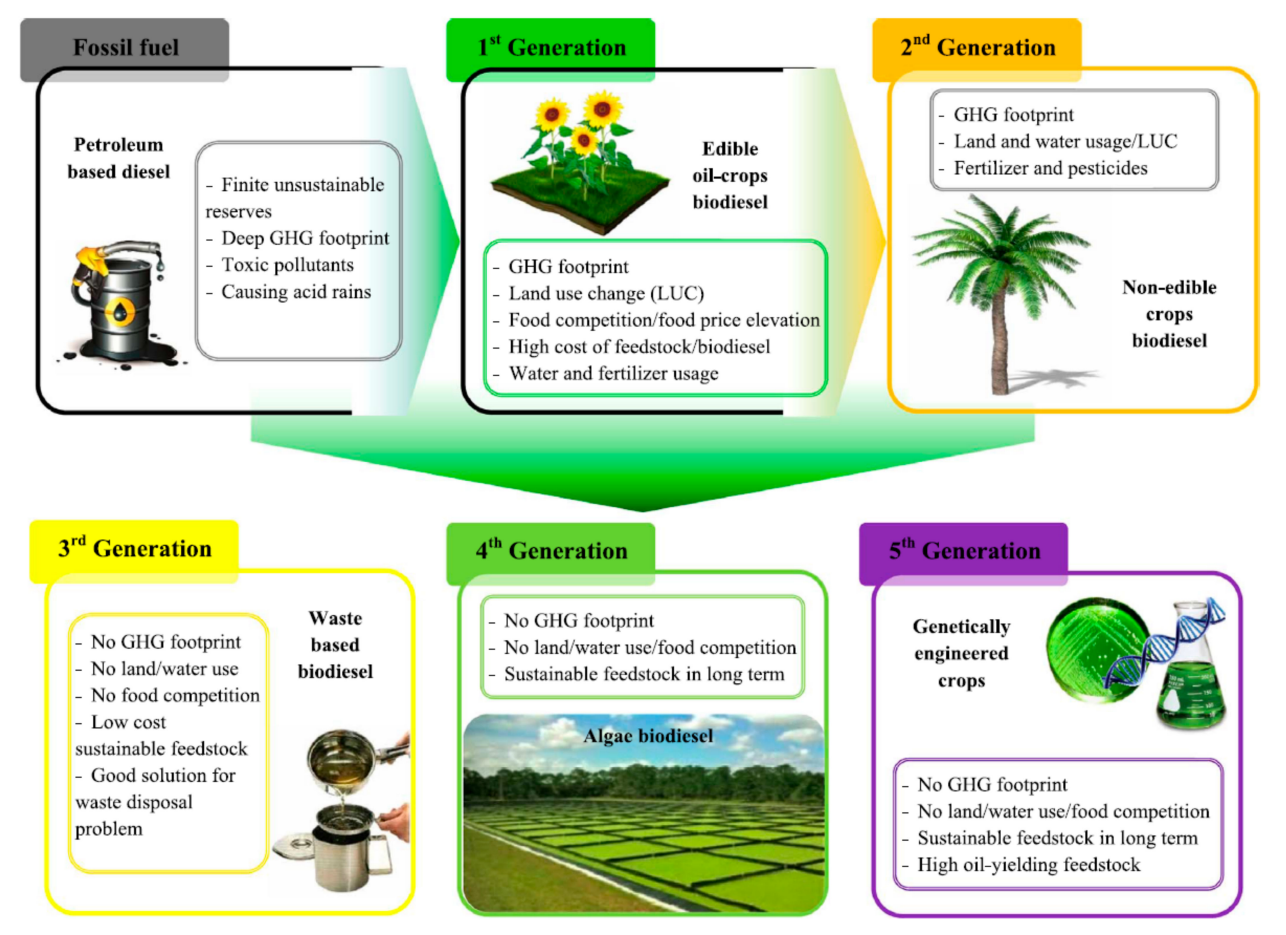
New classification of BD feedstocks based on the sustainability issue. Reprinted with permission from [23]. Copyright (2017) Elsevier B.V.

Conventionally, BD is classified based on their feedstock and production technologies into three different generations, namely, produced from edible oils (1st generation), from non-edible oils (2nd generation), and from other sources such as algae oil, waste cooking oil, and fats (3rd generation) [28]. Hajjari et al. [23] proposed a wider classification of the BD feedstocks, which is based on the sustainability aspects (Figure 5). Taking into consideration that the limiting issue in BD production is feedstock supply, they evaluated the prospects and economic feasibility of the various options of BD manufacturing and noted that the driving force for the migration from food-based biodiesel (first generation) to the next generations had been the fuel vs. food/feed competition over crops, limited arable land and freshwater [61]. In particular, the production of BD from algae is considered to be prospective and economically beneficial [18], and the high relevance of this area of research had been confirmed by a number of actual reviews [62,63,64,65,66,67,68].

To date, more than 95% of biodiesel is produced from edible vegetable oils [69]. However, it can be expected that 2nd and following generations of BD will become more meaningful in the near future—therefore, the study of the CFP of the corresponding BDs and the development of efficient CFIs for novel BD recipes will be performed inevitably.

### 2.2. Synthesis of BD

The basic methods of the synthesis of biodiesel have been reviewed in a number of publications [8,9,10,15,19,24,70,71]. Transesterification of triglycerides with a formation of FA methyl esters (FAMEs) and more expensive and less-used ethyl and isopropyl esters (Figure 2) can be catalyzed by acids and bases. Alkaline catalysts are usually more than three orders of magnitude active in comparison with mineral acids and are commonly applied in industry. Sodium methoxide is one of the most active catalysts for BD production [1]. However, the limited miscibility of triglycerides with methanol hampers the first stage of transesterification in the catalytic synthesis of FAMEs. In addition, high yields of FAMEs can be achieved only if the feedstocks of high purity (the absence of water and hydroxy group containing admixtures) have been used—for example, first-generation edible oils, which brings us back to the problem of fuel vs. food/feed competition.

An efficient method of the synthesis of FAMEs is Supercritical Methanol (SM) processing [70]. Already the first study of Saka and Kusdiana [72] demonstrated the high efficiency of this approach, which was reflected in low reaction time and higher yields of the methyl esters. Further studies of transesterification using supercritical methanol and ethanol confirmed the high efficiency of this approach [73]. Alternative approaches to transesterification of the BD feedstocks use heterogeneous [74,75] and enzymatic [76] catalysts. The rational choice of the catalyst and method of production depends on the type of feedstock.

### 2.3. Chemical Composition of BD

**Figure 6:**
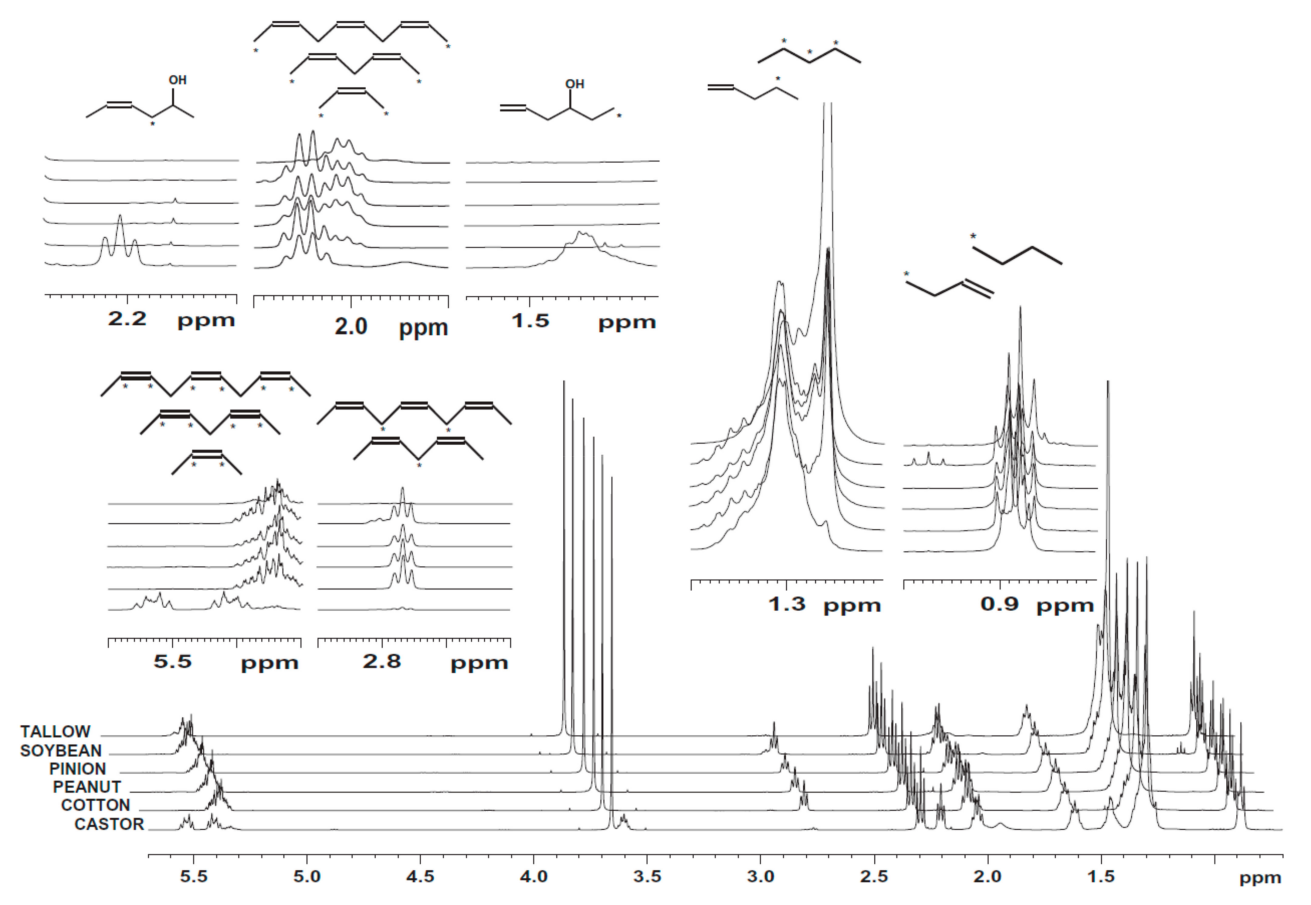
^1^H NMR spectra for pure biodiesel samples from different sources. Reprinted with permission from [82]. Copyright (2012) Elsevier B.V.

Obviously, the chemical composition of the BD is determined by the composition of the feedstocks. The above Table 1 has shown in brief the composition of several major oils in terms of the ratio of FAs in triglycerides. In addition to triglycerides of FAs, BD feedstocks contain components that also have an impact on the CFP of the BD. To date, hundreds of BD compositions obtained from different sources have been analyzed. The chemical analysis of BD is based on gas chromatography (GC) [77,78,79,80,81], GC in combination with mass spectrometry (GC/MS) [80] and NMR spectroscopy [82]. ^1^H NMR spectra [82] (Figure 6) and well-programmed GC/FID [80] allow determining a contence of saturated and poly(unsaturated) FAs in BD samples.

## 3. Cold-Flow Properties of BD

### 3.1. Cold-Flow Properties: Standards and Analysis

The cold-flow properties define the flow behavior of BD at low temperatures. International standards consider three main characteristics, namely, cloud point, pour point and cold filter plugging point. The cloud point (CP) is the temperature of the oil liquid sample when the smallest observable solid particles first occur upon cooling due to crystallization of the alkyl ester with the highest melting point [6]. CP can be measured according to ASTM standards D2500, D5771, D5772, D5773, D6751, D7397 and D7467 [37,83]. CP is related to the possibility of injectors and filters obstruction. The pour point (PP) is the lowest temperature where motion can be detected when the sample is tilted. PP can be measured according to ASTM standards D97, D5949, D5950, D5985, D6749 and D6982 [37]. The cold filter plugging point (CFPP), determined in accordance with ASTM D6371, is the lowest temperature at which a given volume of fuel passes through a standardized wire mesh filter in a specified time. These three characteristics are determined using standard equipment with a significant margin of error.

At the same time, some more knowledge-intensive methods are essential for the study of BD blends and formulations and the investigation of the effect of cold-flow improvers. These methods are differential scanning calorimetry (DSC) or modulated temperature DSC (MT-DSC) [84,85], light scattering [86] and precision rheometry [84,87,88]. The use of these methods allows determining the temperatures of the phase transitions in BD samples with high accuracy.

### 3.2. Cold Flow Properties of BDs: FA Composition

The use of biodiesel is affected by poor cold-flow properties, namely CP, PP and CFPP [6]. These characteristics are essentially concerned with the basic composition and ratio of the fatty acids alkyl esters obtained from different feedstocks. The melting points of the main components of BD were comprehensively studied more than 10 years ago by Knothe et al. [15,89]. The saturated esters have a higher melting point than that of unsaturated esters with the same number of carbon atoms (Table 2); therefore, a higher amount of saturated FA esters in biodiesel fuels can lead to higher CP, PP and CFPP values [90]. A comprehensive study of the CFPP of more than three hundred BD blends was carried out by Yuan et al. [91]. The low-temperature phase behavior of BD components may be complicated by polymorphism and pressure-induced crystallization of FAMEs. To date, very little research has been done on this problem; for example, Liu et al. detected and characterized five crystal modifications of methyl stearate with pressure-dependent stability [92,93], whereas Prathapa et al. studied the crystal package of C8–C13 saturated FAMEs systematically [94].

### 3.3. Microimpurities

In addition to the main components, fatty acid esters, BD can contain microimpurities also affecting the CFP, monoacylglycerols (MAG, formed as intermediates in the transesterification) and steryl glucosides [95]. As was demonstrated by Chupka et al., an increase of 0.01 wt% saturated MAG in a biodiesel blend results in an increase of CP by 4 °C [86]. The high melting points of saturated monoacylglycerols (melting points of monopalmitin and monostearin exceed 70 °C), which can precipitate in different crystal forms [96], and free steryl glucosides (M. P. > 240 °C) cause these materials to crystallize from biodiesel more readily than the esters of fatty acids [34].

## 4. Polymer Additives for BD

Mixing suitable additives to improve the low-temperature characteristics of biodiesel is one of the more effective methodologies. Such additives are known as cold-flow improvers (CFIs) [97,98]. CFIs modify the agglomerative nature of the crystals at low temperatures, as well as transform the crystal shapes from typical plate to needle-like. These smaller crystals do not face viscous resistance while flowing through the fuel filter and injector [99]. Generally, the pour point depressant (PPD) type of CFIs are most effective for biodiesel [100].

The long history of the use of PPDs for the improvement of the cold-flow characteristics of crude oils and petroleum products resulted in the development of various polymeric additives [57,58,59,101]. The use of the polymeric PPDs to upgrade CFP of BD was reported in a number of research papers [97,98,100,102,103,104,105,106,107,108], patents [109,110,111] and raised in recent reviews [33,35,38,39,40,41,54,112]. However, the published data are often fragmentary and sometimes contradictory. In this Section, which in fact is the subject of our review, we tried to summarize and analyze the published data concerning the effect of the polymers of different types on CFP of BDs, obtained from different feedstocks. It is very unfortunate that a number of publications did not include the data concerning the composition and molecular weight characteristics of polymeric PPDs under study as opposed to PPDs synthesized and studied in the improvement of the CFP of crude oils and petroleum-based products.

### 4.1. Polyolefins

Polyolefin CFIs, based on polyethylene-poly(ethylene-*alt*-propylene) [113,114,115,116], ethylene/1-butene or ethylene/higher α-olefin copolymers [117,118,119,120], demonstrated marked efficiency in the improvement of the CFP of petroleum-based products. However, similar polymers did not show a substantial effect when used in BDs. For example, a number of industrial polyolefin depressants were tested in 0.1 wt% concentrations by Nie and Cao as CFIs for waste cooking oil BD [108]. In all cases, polyolefin PPDs demonstrated moderate efficiency, the lowering of PP and CFPP was 2–3 °C. Note that comonomer composition and ratios, as well as molecular weight characteristics of polyolefin PPDs, used in [108], have not been reported in detail. Commercial polyolefin depressant T803 (Antai Chemicals Co. Ltd., Shanghai, China) was tested as 0.02–0.08 wt% solutions in waste cooking oil BD [98], the lowering of CP and CFPP was only 1 °C, the maximum impact on PP was −3 °C when 0.04 wt% of T803 was added.

Sern et al. reported no effect of poly(1-decene) for palm oil BD up to 2 wt% concentrations of the additive [105].

### 4.2. Ethylene/Vinyl Acetate Copolymers

**Scheme 1:**
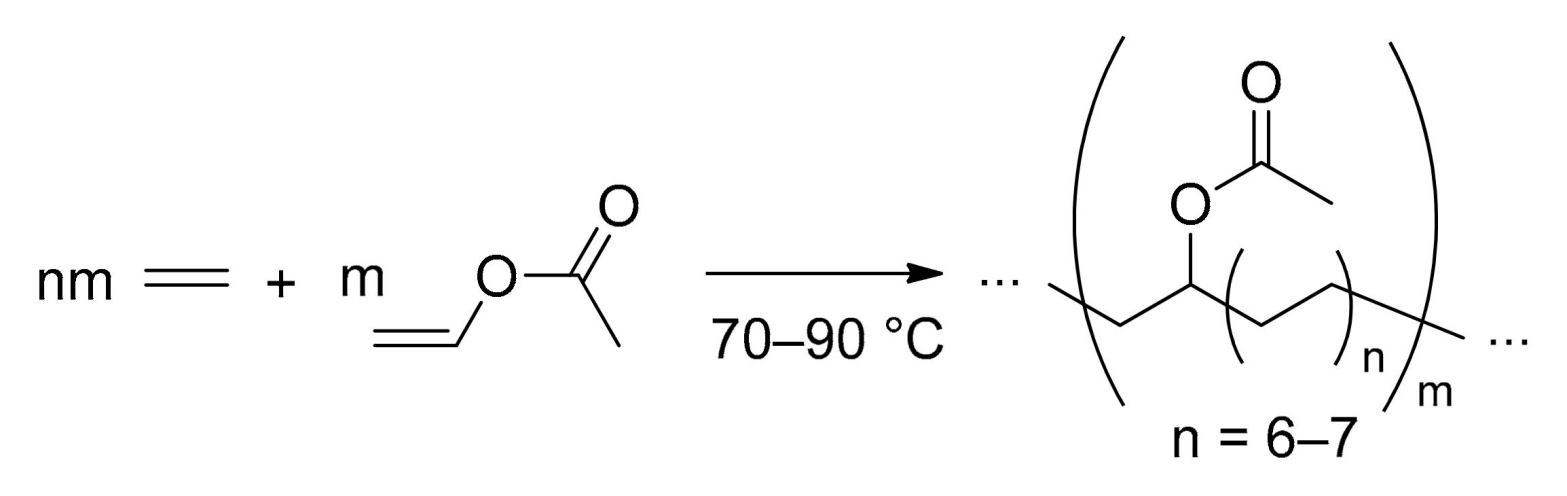
The synthesis of ethylene/vinyl acetate copolymers (EVA).

Copolymers of ethylene and vinyl acetate (EVA copolymers) represent bulk products that are manufactured using high-pressure free-radical copolymerization of low-cost comonomers, ethylene and vinyl acetate (Scheme 1) and represent the most widely used PPDs for petroleum-based diesel fuel [121,122,123].

The effectiveness of EVA copolymers very much depends on the comonomers ratio, the optimum ratio of ethylene and vinyl acetate is 6–7 for copolymers used for CFIs of petroleum diesel [124]. The mechanism of the effect of EVA copolymers on the paraffin deposition in petroleum diesel is attributable to the coordination of the oleophilic fragment of the EVA copolymer macromolecule on the surface of the paraffin crystals resulting in the formation of smaller-scale crystals [125]. Moreover, the same mechanism is applicable for BDs (see below).

Wang et al. reported that EVA copolymers have no effect on CP of waste cooking oil BD at concentrations of 0.02–0.08 wt%, CFPP was reduced by 2 °C; however, the lowering of PP was more visible, up to 6 °C [98]. For soybean BD, the presence of EVA copolymer in 0.01 wt% concentration resulted in PP lowering by 2 °C [106]. Gao et al. studied CFP of the waste cooking oil BD at EVA copolymer concentrations of 0.02–0.08 wt% and detected only a marginal effect (no change of CP, the lowering of PP by 3 °C) [103]. For canola-based methyl esters, the addition of 1 wt% EVA copolymers resulted in lowering of PP by 11 °C, but CP remains unchanged [100]. The addition of 0.3 wt% of the commercial additive Keroflux (BASF), ethylene/vinyl acetate/acrylate copolymer (86, 16 and 3 mol%, respectively, *M*_n_ = 4.14 kDa) to the blend of rapeseed and soybean oil methyl esters reduced CP by 2 °C and CFPP by 9 °C [126].

**Figure 7:**
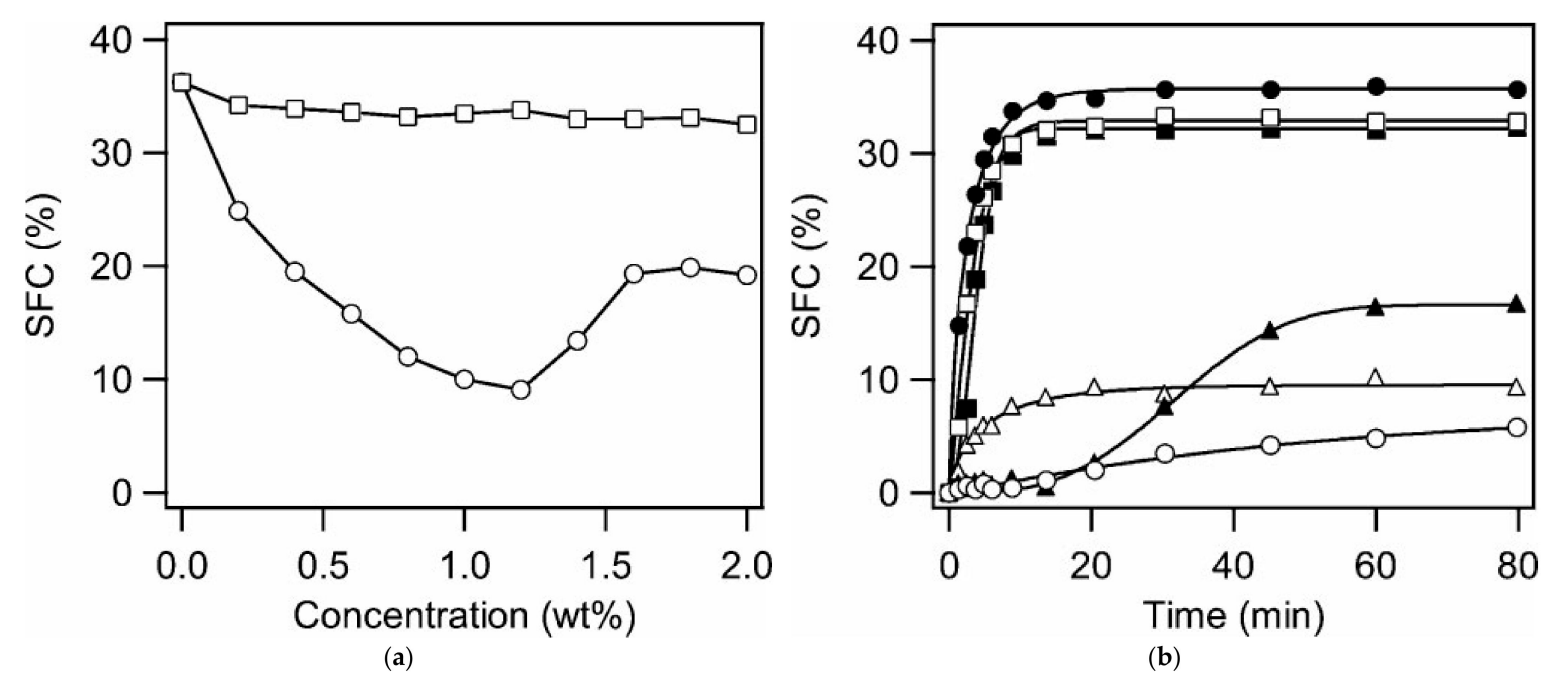
(**a**) Solid Fat Content (SFC) NMR analysis for PGE and EVA additives (2 wt%, open circles and open squares, respectively) taken at 9 °C after 60 min; (**b**) Time variation of SFC with and without additives measured at 9 °C. Pure PFME (closed circles), 1 wt% EVA (open squares), 2 wt% EVA (closed squares), 1 wt% PGE (open triangles), 2 wt% PGE (closed triangles) and 1 wt% PGE + 1 wt% EVA (open circles). Reprinted with permission from [127]. Copyright (2010) Wiley-VCH Verlag GmbH & Co.

Hamada et al. evaluated polyglycerol esters of fatty acids (PGE) and EVA copolymer as additives to improve the CFP of palm oil-based BD (46% of saturated FAMEs, CP = 15 °C, PP = 14 °C) [127]. They found that these additives led to the formation of considerably small and fine-dispersed crystals, but such an effect was not observed when EVA copolymer was used alone. The synergy in the simultaneous addition of PGE and EVA copolymer was convincingly demonstrated by using Solid Fat Content (SFC) NMR analysis of the direct ratio between the solid and liquid parts of the sample (Figure 7). Figure 7a clearly illustrates the low efficiency of the EVA copolymer; however, an addition of PGE significantly decreased the SFC values. The evolution of SFC values over time under isothermal conditions (Figure 7b) showed that the simultaneous addition of 1 wt% EVA copolymer and 1 wt% PGE results in a dramatic decrease in SFC values.

**Figure 8:**
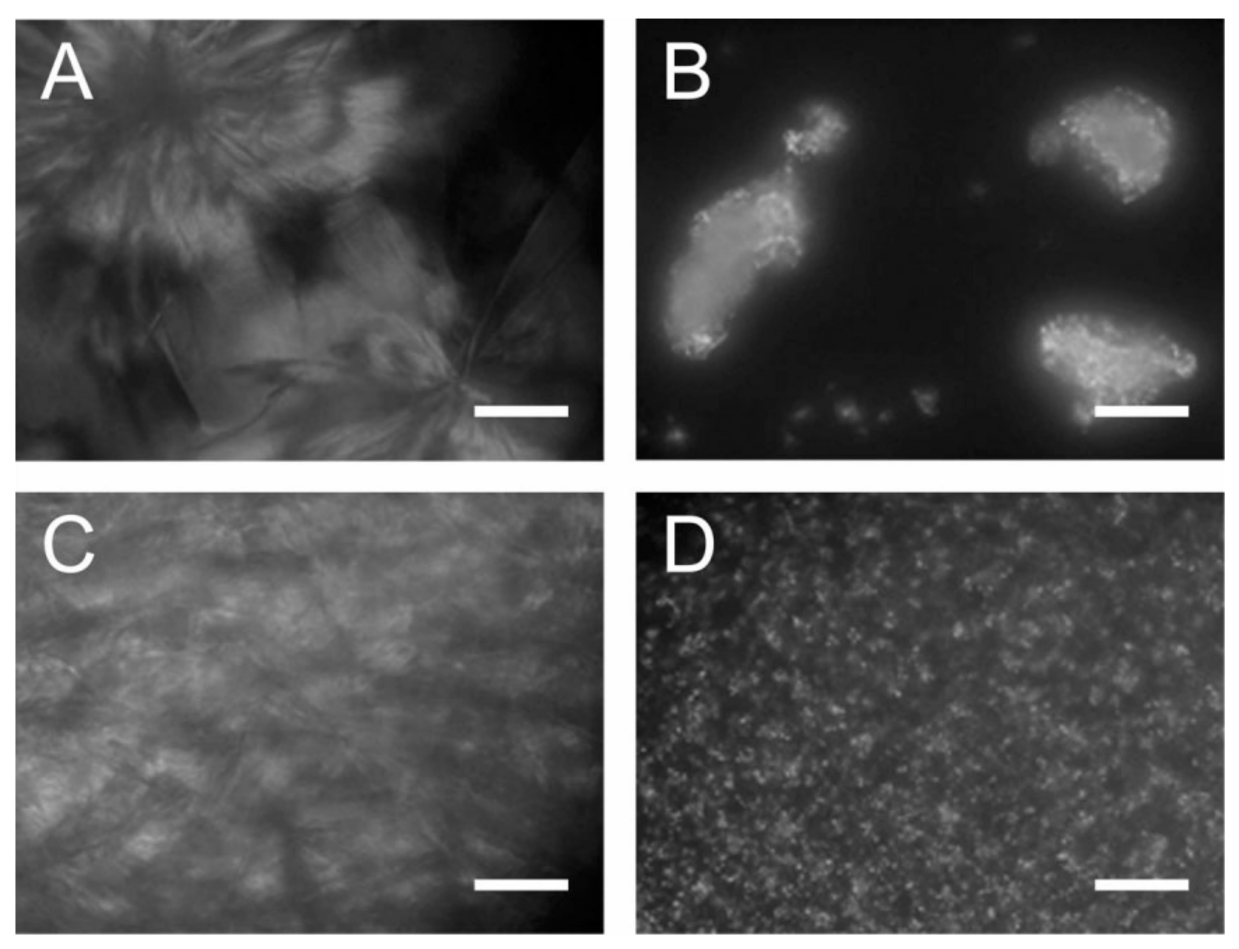
Optical micrographs of PFME crystals taken during the cooling process. Scale bar indicates 100 μm. (**A**)No additives, (**B**) 2 wt%PGE, (**C**) 2 wt% EVA and (**D**) 1 wt% PGE þ 1 wt% EVA. Reprinted with permission from [127]. Copyright (2010) Wiley-VCH Verlag GmbH & Co.

Drastic changes in crystal morphology were noticed by Hamada et al. [127] using polarized light microscopy. Figure 8 shows the crystals observed at the crystallization temperature *T*_C_ when the sample was slowly cooled from 25 °C to 6 °C. Large bulky crystals were observed in the absence of additives at 13 °C (*T*_C_ of pure BD, Figure 8A). In the presence of 2 wt% PGE, the size of crystals was reduced to 100 μm, the crystallization was observed at 8 °C (Figure 8B). An addition of 2 wt% EVA formed crystal spherulites (Figure 8C), which were composed of needle-like crystals. Finally, the simultaneous addition of 1 wt% EVA copolymer and 1 wt% PGE caused the formation of small crystals (Figure 8D). For the last case, CFPP was reduced by the value of 6 °C.

**Scheme 2:**
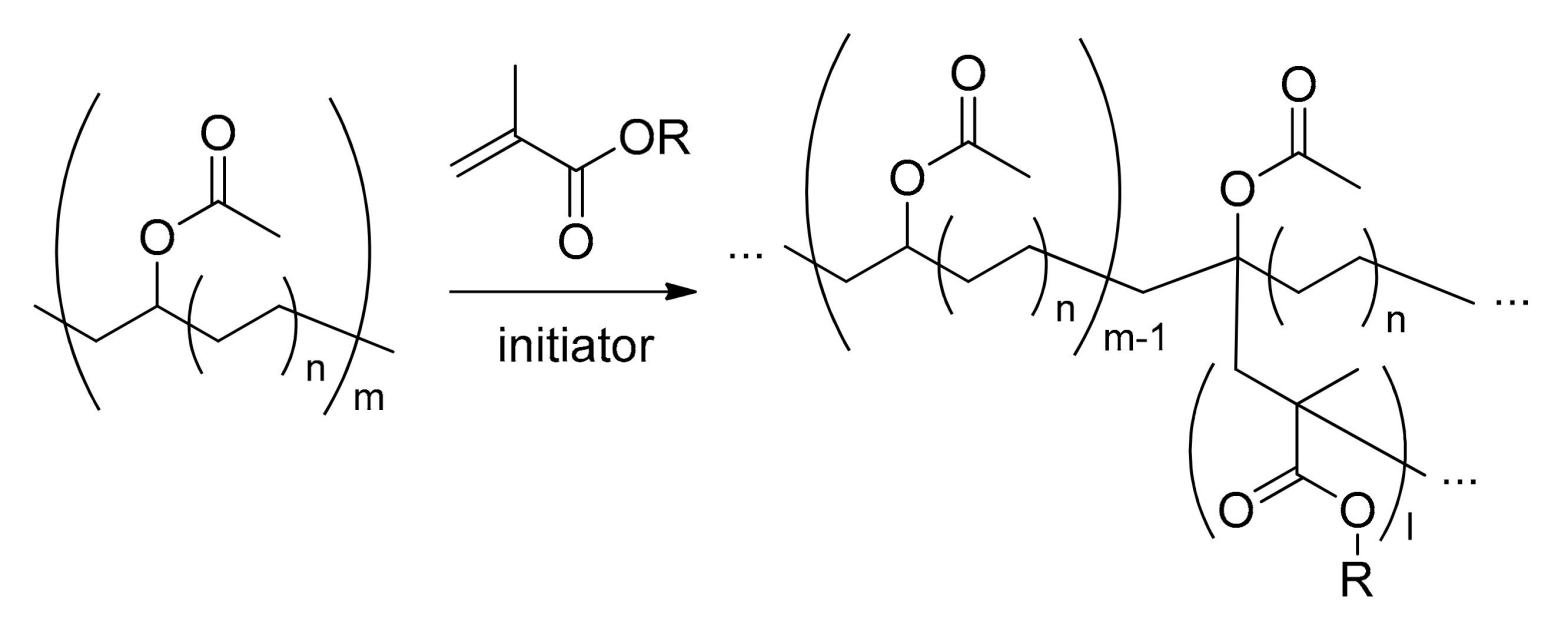
The synthesis of methacrylate-grafted EVA copolymers.

The effectiveness of EVA copolymers as pour point depressants can be substantially improved by free-radical grafting using alkyl methacrylates (Scheme 2) [128,129]. This reaction was used for the modification of partitional hydrolyzed EVA copolymers [130]. The effectiveness of grafted EVA copolymers was studied for mixtures with poly (methacrylates); see Section 4.5.

### 4.3. Polyacrylates and Related Copolymers

**Scheme 3:**
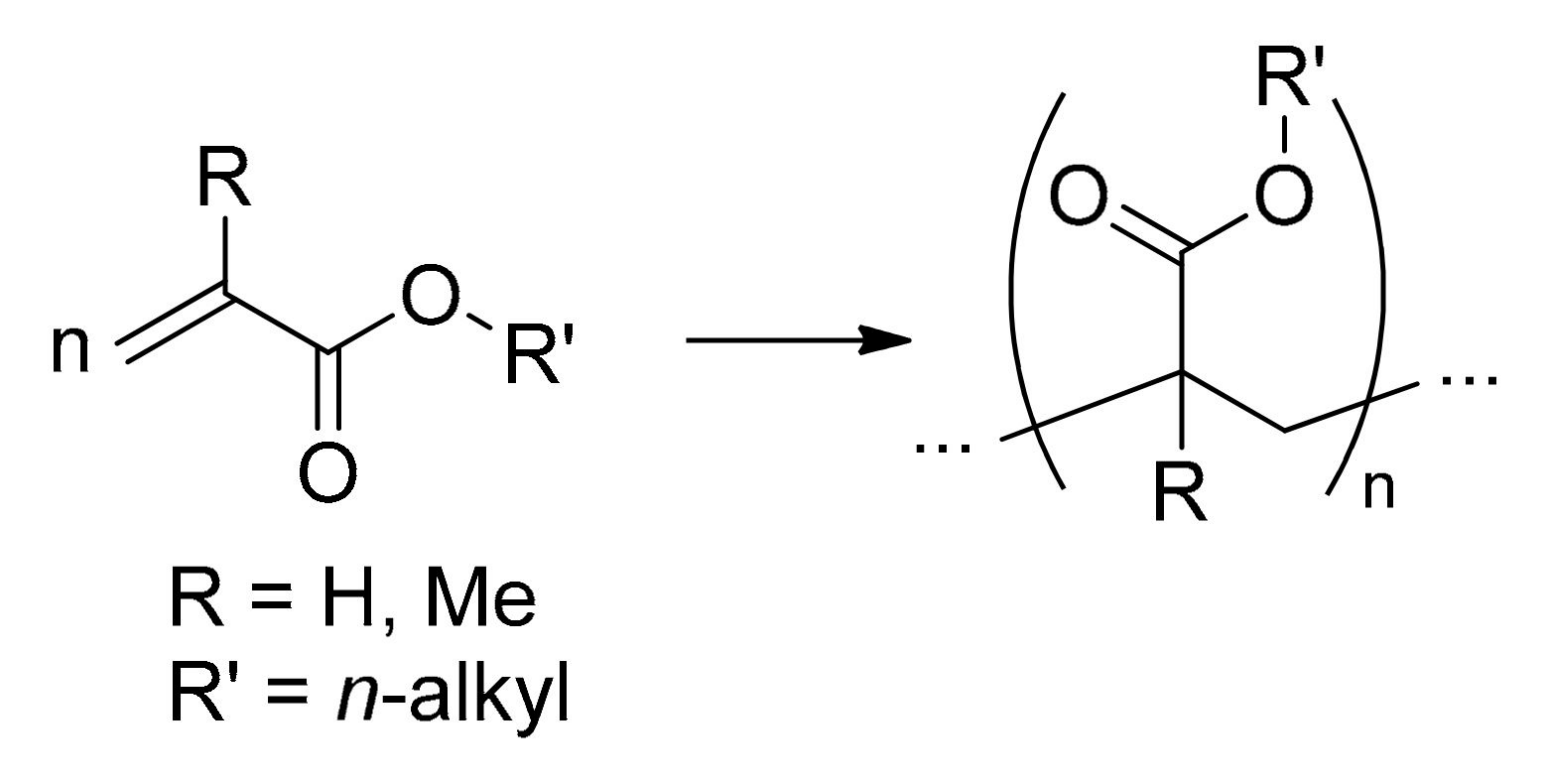
The synthesis of poly((meth)acrylate)s.

(Co)polymers of the esters of acrylic and methacrylic acids are typically obtained by free-radical (co)polymerization (Scheme 3). The effectiveness of these CFIs in lowering of CP, PP and CFPP of the petroleum diesel is directly related to the length of the alkyl fragments in these esters, and the best results were obtained for the derivatives of C_18_ and higher alcohols [131,132].

The study of the impact of poly(meth)acrylates and other polymeric additives on CFP of canola oil-based methyl esters (canola BD, 6.2 wt% of saturated fatty acids, PP = −16 °C) was conducted by Chastek [100]. He demonstrated that poly(octyl methacrylate) (*M*_n_ = 100 kDa) and poly(decyl methacrylate) (*M*_n_ = 100 kDa) are ineffective as pour point depressants for canola BD. Poly(dodecyl methacrylate) (*M*_n_ = 250 kDa), on the other hand, showed a remarkable decrease of PP being used in 0.5–1 wt% concentrations. When 1 wt% was used, the PP of canola BD was −46 °C. Poly(hexadecyl methacrylate) (*M*_n_ = 200 kDa) proved to be ineffective (PP was −20 °C for 1 wt% concentration).

Muniz et al. studied the effect of the addition of 0.01–0.1% poly(alkyl acrylates) (alkyl = *n*-C_8_H_17_, *M*_n_ = 4.3 kDa, *Ð*_M_ = 1.03; *n*-C_12_H_25_, *M*_n_ = 4.0 kDa, *Ð*_M_ = 1.04; *n*-C_14_H_29_, *M*_n_ = 4.4 kDa, *Ð*_M_ = 1.01) and poly(alkyl methacrylates) (alkyl = *n*-C_8_H_17_, *M*_n_ = 4.0 kDa, *Ð*_M_ = 1.03; *n*-C_14_H_29_, *M*_n_ = 3.9 kDa, *Ð*_M_ = 1.04) to palm oil BD [107]. In all cases, the lowering of PP was negligible.

**Figure 9:**
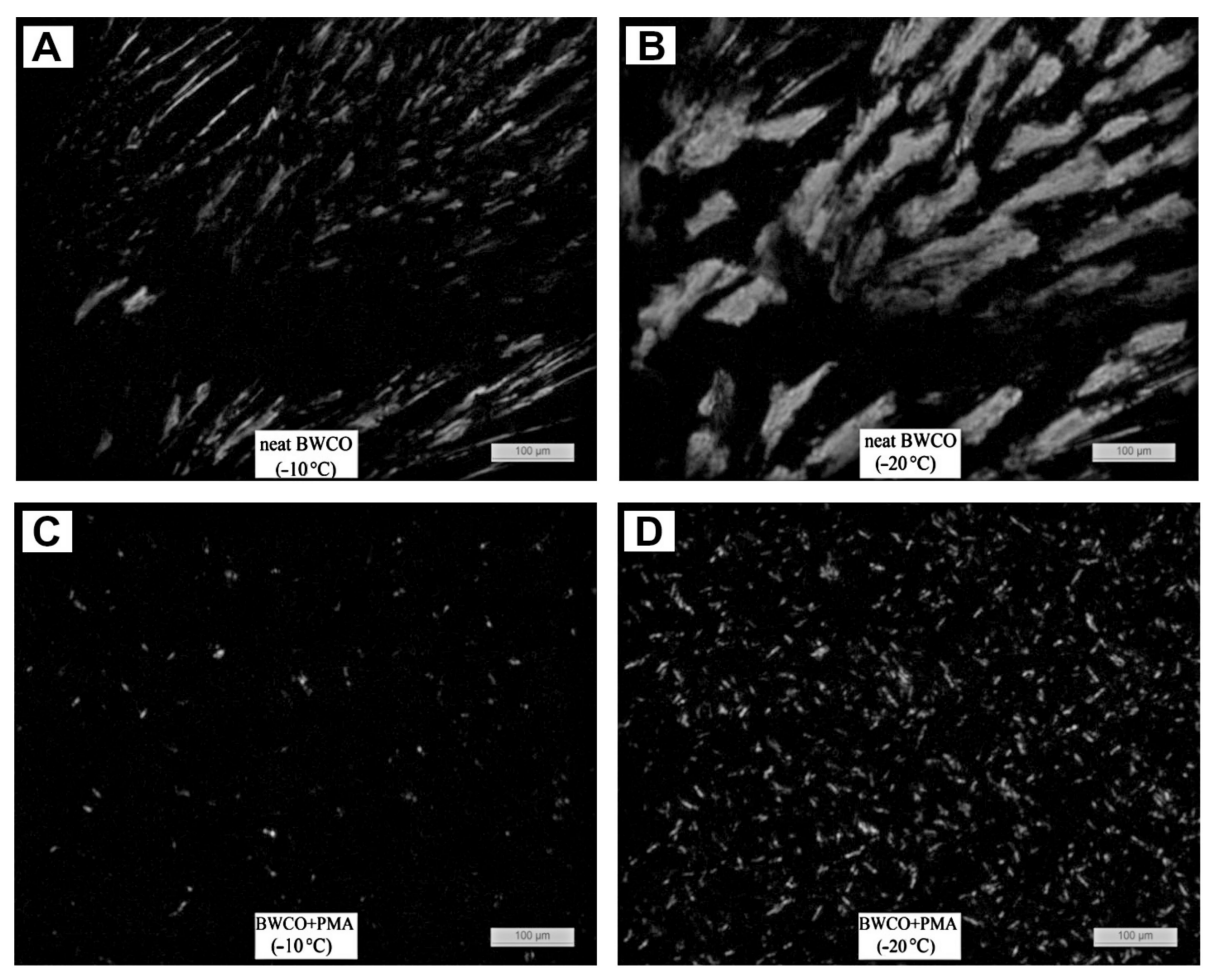
Polarizing optical microscopy images of crystal morphologies of waste cooking oil BD without or with polymethyl acrylate: crystallization of saturated FAMEs without additive at –10 °C (**A**) and–20 °C (**B**), and with additive at –10 °C (**C**) and–20 °C (**D**). Reprinted with permission from [98]. Copyright (2014) Elsevier B.V.

In a comparative study of different polymeric CFIs [98], the commercial depressant A-4 (Shanghai Hailian Lubricating Material Technology Co. Ltd., Shanghai, China) was presented as “polymethyl acrylate” without a more detailed description. This polymer did not affect CP of the waste cooking oil-based methyl esters at concentrations of 0.02–0.08 wt% but reduced PP by the value of 8 °C. It is noteworthy that the maximum of PP depression was detected for 0.04 wt% concentration of the additive, and a further increase of the concentration of polyacrylate resulted in an increase of PP. This concentration of the additive was also optimal for CFPP decreased by 6 °C. Polarizing optical microscopy images for neat waste cooking oil BD and BD treated with 0.04 wt% polymethyl acrylate are presented in Figure 9. In polarizing microscopy analysis, crystals reflect polarized light (white images), amorphous substances absorb light (black images). In neat BD, large crystalline aggregates formed rapidly on cooling from −10 °C to −20 °C (Figure 9A,B). The presence of polymethyl acrylate revealed the formation of small amounts of crystals at −10 °C (Figure 9C), and at −20 °C, some tiny needle-like crystals were formed (Figure 9D). Thus, polymethyl acrylate effectively prevented the aggregation of crystals and transformed the crystal shape. Therefore, lowering PP and CFPP of BD acrylates demonstrated the mechanism of wax crystal modification, similar to the mechanism of the action of EVA copolymers. During the study of the effect of commercial poly(methacrylate) 10-320 (Evonik Oil Additives GmbH, Essen, Germany) on CFI of soybean biodiesel [133], this mechanism was confirmed.

The efficiency of poly(methacrylate) pour point depressant T602 (Tuofu Lubricating Oil Additive Co., Ltd., Hebei, China) was studied by Xue et al. in waste cooking oil BD (32 wt% of saturated FAMEs, CP = 7 °C, PP = 5 °C) [134]. When T602 (0.5 wt%) was used in combination with Span80 dispersant (0.25 wt%), the best synergistic effect on reducing the CFPP by 7 °C was detected. Commercial pour point depressant Wintron Synergy (Biofuel Systems Group LTD, Ormskirk, UK), which is a combination of polymethacrylate compounds in a solution of mineral oil, demonstrated CP decrease of canola oil methyl esters by 5 °C when 2 wt% of the additive was used [135].

The use of poly(acrylate) CFIs was also presented in the patent literature. 0.5–1 wt% of poly(alkyl methacrylate)s, prepared from C_12_–C_18_ linear alcohols, demonstrated reduction of PP for soybean oil methyl esters up to 12 °C, and for palm oil methyl esters up to 9 °C [110]. The effect of the polyacrylates on biodiesel with low content of saturated fatty acids (~10%, rapeseed oil methyl esters or 1:1 blend of rapeseed and soybean oil methyl esters) was studied by Mähling et al. [126]. For 0.1 wt% concentration of poly(dodecyl-pentadecyl methacrylate) (*M*_n_ = 3.74 kDa, *Ð*_M_ = 1.54), the lowering of PP and CFPP was 3 °C. For 0.3 wt% concentration of the copolymer of stearyl methactylate and lauryl methacrylate (*M*_n_ = 8.5 kDa, *Ð*_M_ = 1.72), the lowering of PP was 9 °C; the maximum reduction of CFPP (6 °C) was detected when this copolymer was used in 0.5 wt% concentration [126]. For soybean oil BD (CP = −2.5 °C, PP = −5 °C), the addition of 0.1 wt% of poly(lauryl acrylate)-*b*-poly(lauryl acrylate/stearyl methacrylate) reduced the PP by 5 °C [136].

**Scheme 4:**
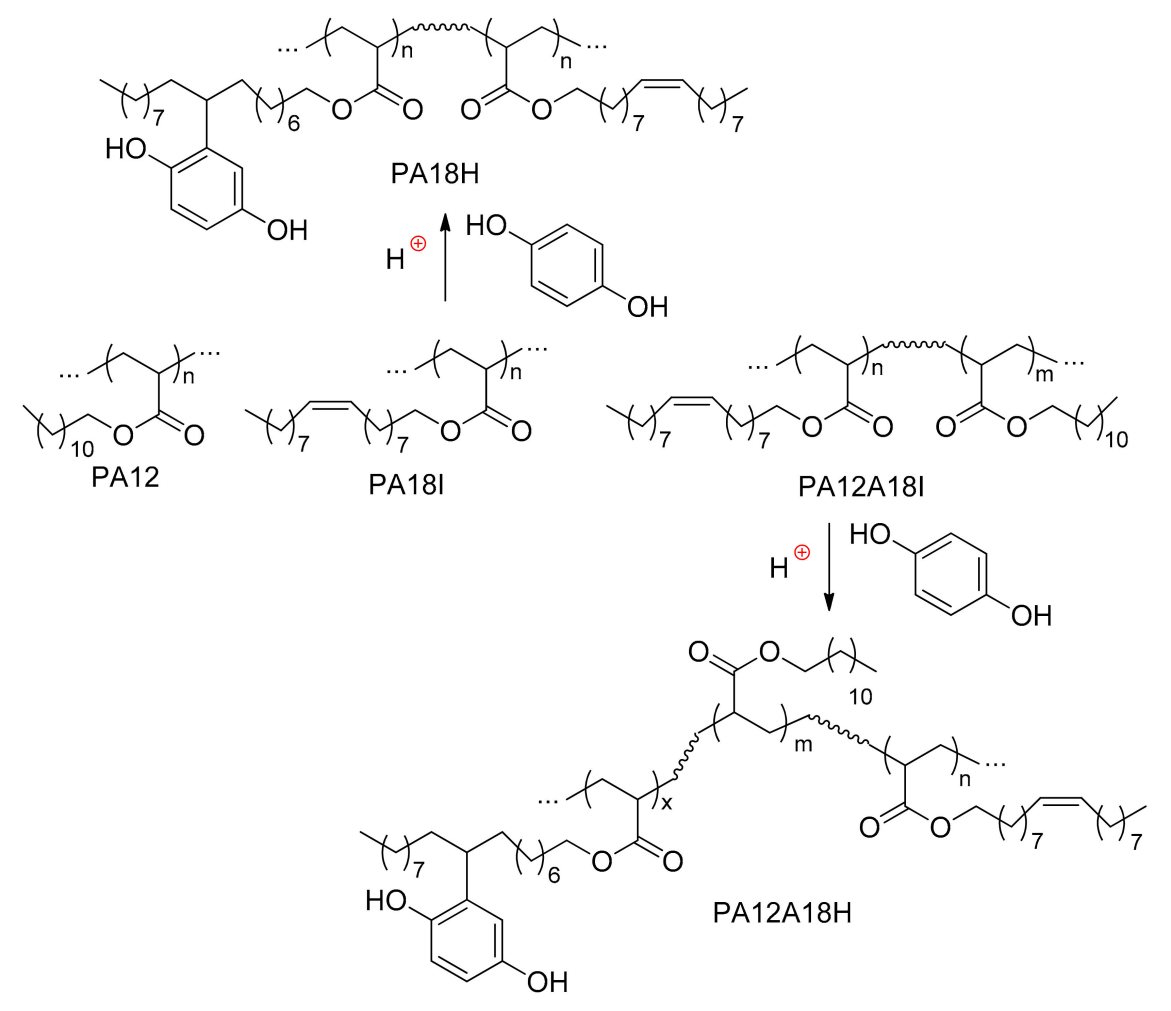
Structures of poly(acrylates) and the preparation of hydroquinone-functionalized polyacrylates [137].

**Figure 10:**
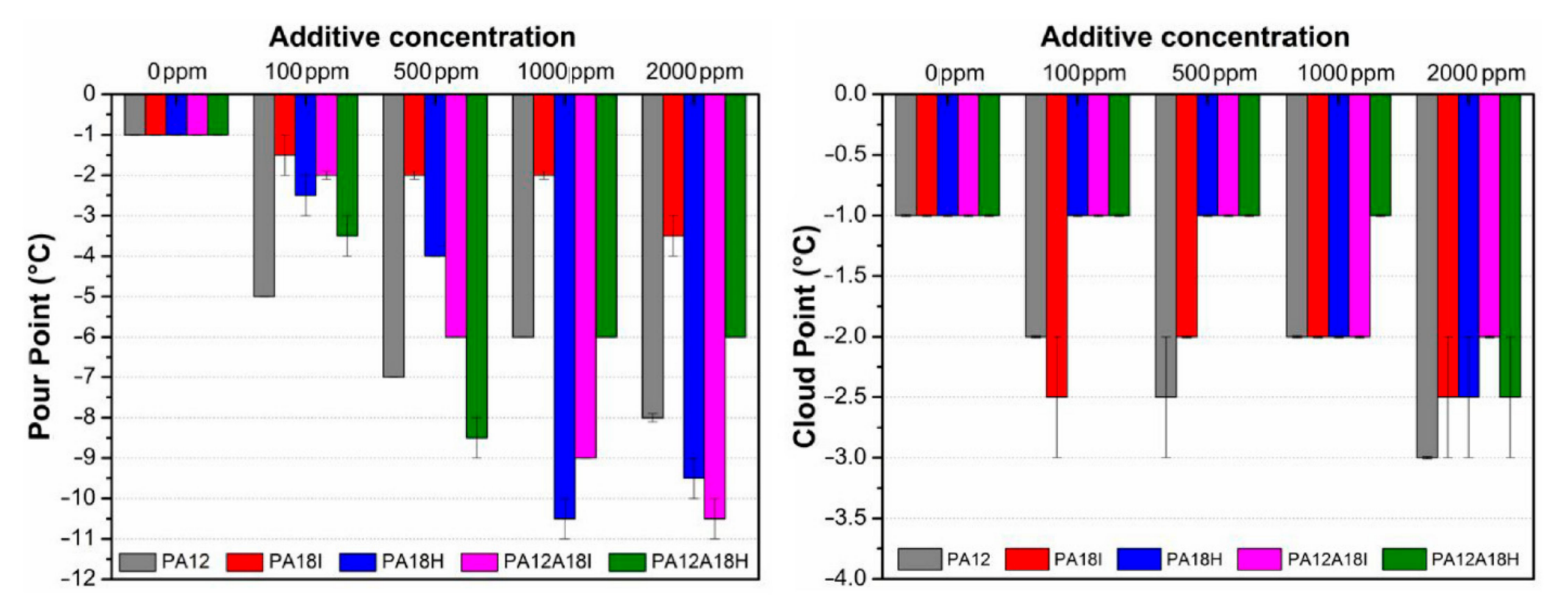
PP (**left**) and CP (**right**) of soybean oil BD with and without additives. Reprinted with permission from [137]. Copyright (2020) American Chemical Society.

The recent work of Muniz et al. [137] deserves special mention. To improve the CFP of soybean oil BD (CP = −1 °C, PP = −1 °C), they synthesized a series of polyacrylates that contained oleyl or/and dodecyl fragments; unsaturated copolymers were then functionalized by acid-catalyzed reactions with hydroquinone (Scheme 4). The effect of these additives on the CFP of soybean oil BD is presented in Figure 10.

Mohanan et al. [54] demonstrated a marked synergistic effect of the use of poly(lauryl methacrylate) as PPD in combination with low molecular weight crystallization modifiers, derivatives of vegetable oils. In such mixtures, crystallization modifiers delay the crystallization and form crystals with surface characteristics most favorable for adsorbing the PPD. As a result, these complex additives not only affect the nucleation and crystal growth but also provide a barrier to agglomeration, thus preventing the gelling of the biodiesel for an extended temperature range. PP depression for soybean oil methyl esters reached 30 °C for poly (lauryl methacrylate) loading of 0.5 wt%.

Note that Sern et al. reported no effect of the poly (octadecyl methacrylate) additive for palm oil BD up to 2 wt% concentrations [105].

### 4.4. Maleic Anhydride Copolymers and Their Derivatives

**Scheme 5:**
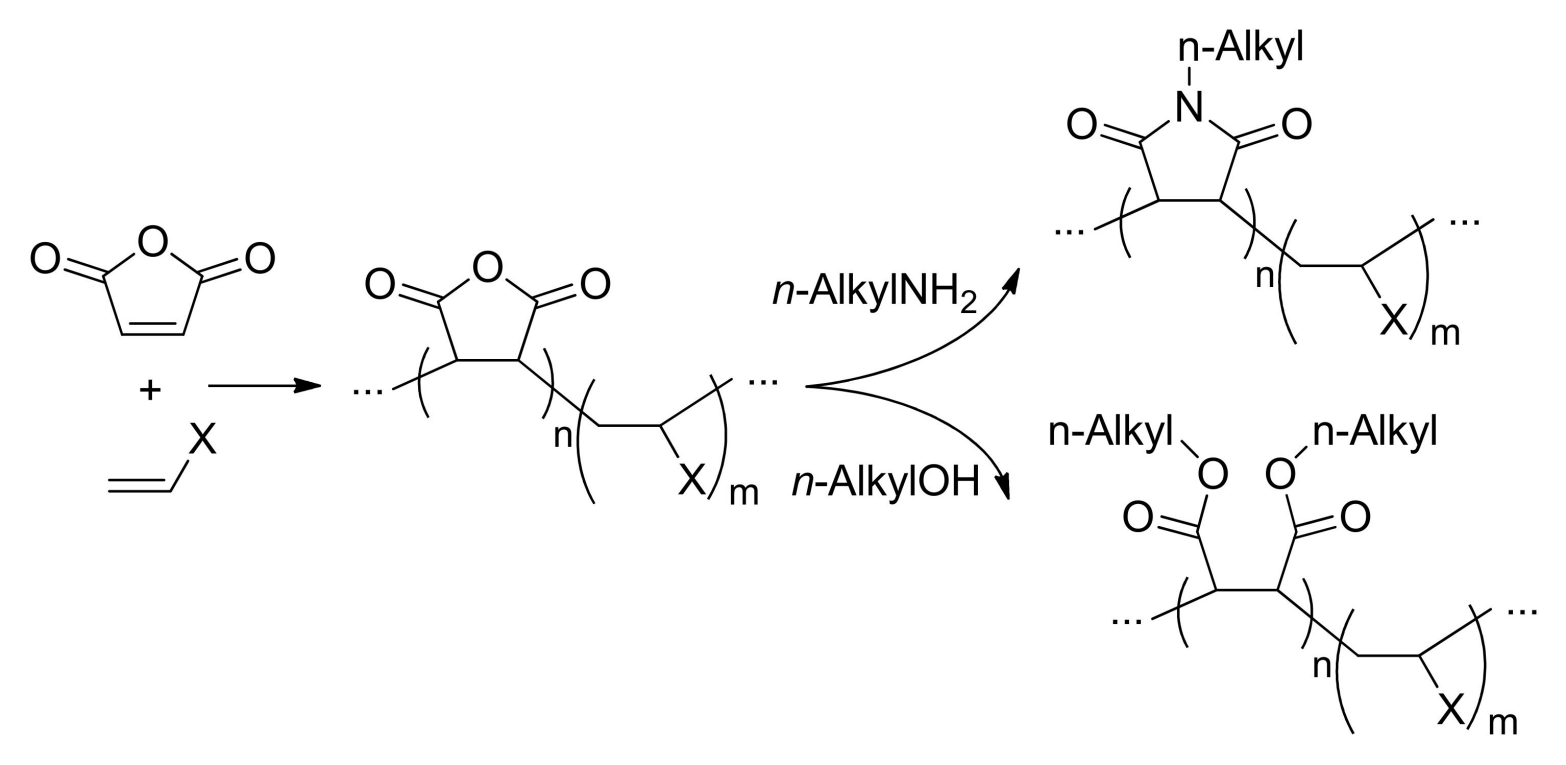
The synthesis and further modification of maleic anhydride copolymers.

Copolymers of maleic anhydride (MA) with different vinyl substrates are highly attractive due to the possibility of the further modification of the succinic anhydride fragments formed during polymerization. Usually, such modification is imidization or esterification (Scheme 5).

Copolymers of MA with C14, C16 and C18 *n*-alkyl methacrylates were synthesized and modified by the reaction with tetradecylamine [104]. These copolymers were studied as CFIs for waste oil biodiesel. For 0.05 wt% concentrations of additives, the lowering of PP was 13 °C, 12 °C and 10 °C for imide-functionalized copolymers of MA with C14, C16 and C18 *n*-alkyl methacrylates, respectively.

Copolymers of MA with donor monomers are usually attributed to alternating copolymers [138,139,140,141,142,143,144], and the preference of the formation of alternating sequences in free-radical copolymerization of MA with different olefins was confirmed by the density functional theory (DFT) simulations [145]. The wide spectra of the functional derivatives of MA copolymers are considered as highly efficient depressor additives and flow improvers for petroleum fuels [146,147,148].

Poly(MA-*alt*-1-octadecene) reduced PP of palm oil BD by 6 °C being used in 2 wt% concentration [105]. For tobacco seed oil (12 wt% of saturated FAMEs), the use of 1 wt% of poly(MA-*alt*-1-octadecene) reduced CFPP by the value of 7 °C [149]. The copolymer of MA with octadecyl vinyl ether (*M*_n_ = 7.0·10^3^ Da) in 1 wt% concentration lowered PP of canola BD by 3 °C [100].

The products of the reaction of poly(MA-*alt*-C_18_H_36_) (*M*_n_ 3300 Da) with a mixture of tetradecanol, BnOH and C_12_–C_18_ aliphatic amines [150] in 100 ppm concentration reduced CP of BD by 5 °C. Tasić et al. reported that MA-olefin-acrylate copolymers, functionalized by aliphatic amides, demonstrate marked efficiency as CFIs for rapeseed oil BD [151]. However, this work, like many other articles, did not include a detailed description of the copolymer used.

**Scheme 6:**
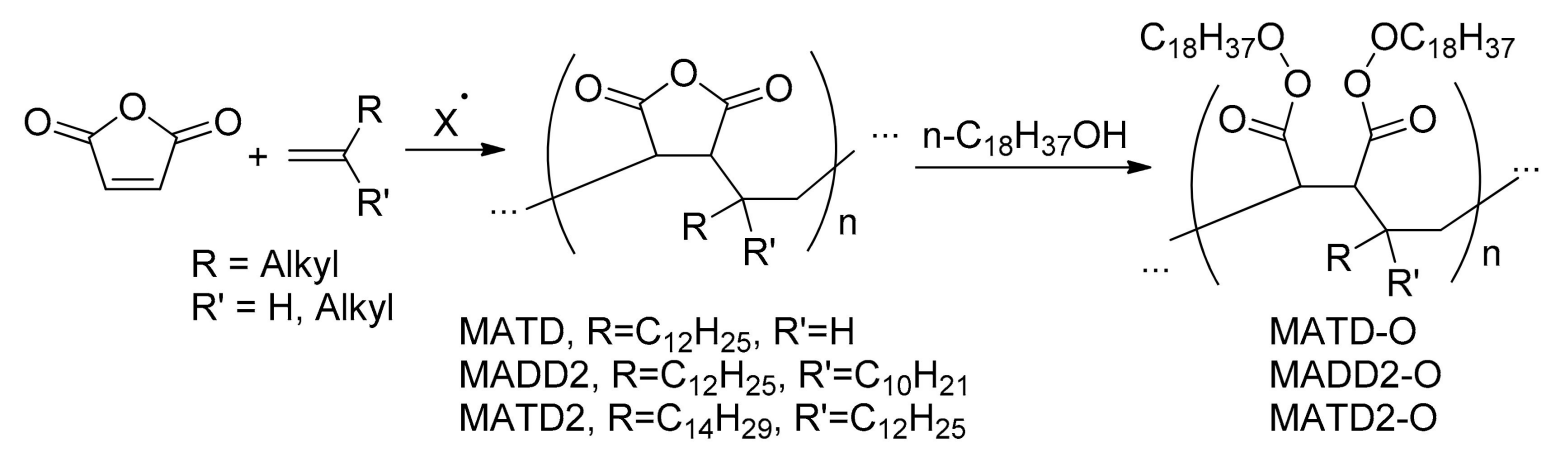
Copolymerization of maleic anhydride (MA) with olefins and functionalization of poly(MA-*alt*-olefin)s [152].

Very recently, we made a comparative study of the effectiveness of a number of conventional CFIs of the EVA copolymer family and octadecanol-functionalized MA copolymers with tetradecene and vinylidene dimers of 1-dodecene and 1-tetradecene (Scheme 6) [152].

**Figure 11:**
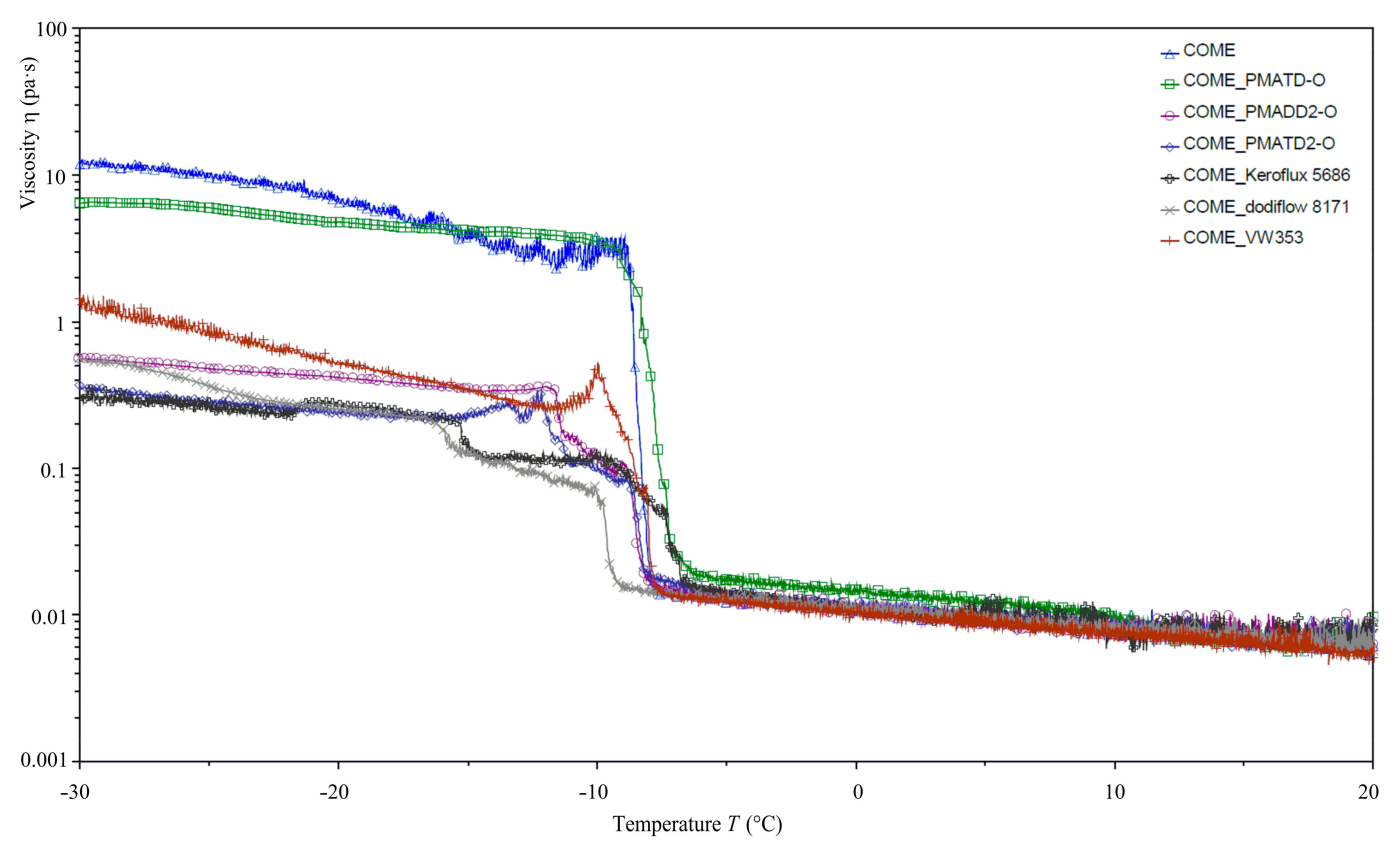
Viscosity vs. temperature curves for corn oil biodiesel (corn oil methyl esters, COME) and COME containing 0.1 wt% polymer additives. Reprinted with permission from [152]. Copyright (2021) Elsevier B.V.

Using precision rheometry (Figure 11) and DSC, we demonstrated that functionalized MA copolymer with 1-tetradecene dimer demonstrated promising characteristics as CFIs for corn oil BD (23% of saturated FAMEs). Note that high effectiveness was observed for maleic anhydride copolymer with long-chain branched hydrocarbon, dimer of 1-tetradecene. Additionally, note that the standard methods of determining CP and PP could not detect subtle differences in biodiesel rheology at subzero temperatures, a proper comparison of the effectiveness of polymer additives requires the use of more knowledge-intensive methods such as precision rheometry and DSC.

### 4.5. Other Polymeric CFIs

Promising results were obtained for the mixtures of methacrylate grafted EVAs and poly(methacrylates) [126]. Addition of 0.3 wt% of 1:1 mixture of dodecyl-pentadecyl methacrylate grafted EVA (*M*_n_ = 51.17 kDa, *Ð*_M_ = 2.14) with poly(dodecyl-pentadecyl methacrylate) (*M*_n_ = 3.74 kDa, *Ð*_M_ = 1.54) to 1:1 blend of rapeseed and soybean oil methyl esters resulted in reducing of CFPP and PP by the values of 5 °C and 15 °C, respectively.

**Scheme 7:**
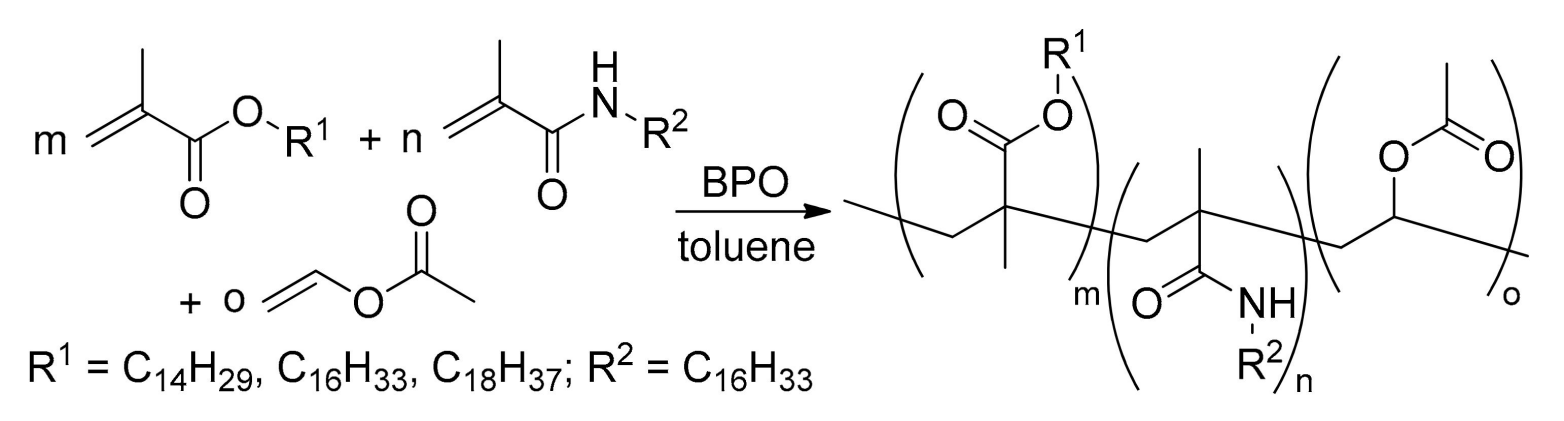
The synthesis of methacrylate/methacrylamide/VA terpolymers [108].

A number of terpolymers were synthesized by free-radical copolymerization of alkyl methacrylates, *N*-hexadecylmethacrylamide and vinyl acetate using benzoyl peroxide (BPO) as an initiator [109] (Scheme 7). At a high concentration of the additive (0.5 wt%), the lowering of PP and CFPP was less than 5 °C.

Tesfaye and Katiyar studied oligomers of l-lactic acid (*M*_n_ ~ 1 kDa) as CFIs for soybean oil BD (CP = 4 °C, PP = −2 °C) and established only limited effect even at 2 wt% concentration of the additive [153]. It is significant, however, that low-MW poly(l-lactide) substantially reduced the CP of the soybean oil BD (to 0 °C) without affecting the PP.

## 5. Polymer Additives for BD/Petroleum Diesel Blends

Obviously, strictly BDs represent fuels with not good cold-flow properties. Separation of the components with high melting points (winterization, [6,90,154]) considerably complicates BD production and makes this process more expensive. To date, the blending of BDs with petroleum diesel is the most efficient and commonly used approach to involve BDs in the global fuel industry [11,17,20,21]. However, even several per cent of the esters of saturated fatty acids have a negative effect on CFP. The improvement of the CFP BD/petroleum diesel blends is certainly an actual problem.

The effect of polyolefin PPDs (copolymers of C_9_–C_22_ α-olefins) on CFP of waste cooking oil BD blends with petroleum diesel was studied by Xue et al. [102]. They demonstrated that polyolefin additive is effective cold-flow improver for the mixture of BD (25% of saturated FAMEs, CP = 7 °C, PP = 5 °C) with petroleum diesel (CP = −1 °C, PP = −10 °C), containing 20 vol% of BD. For optimal concentration (0.04 wt%), the lowering of CP and PP were 8 °C and 7 °C, respectively. In addition, the presence of polyolefin additives substantially reduced the low-temperature viscosity of BD/petroleum diesel blends.

The results of the first comparative study of the effect of different additives on CFP of soybean BD/petroleum diesel blends were reported by Chui et al. [97]. The optimum concentrations of the additives were found to be 0.2–0.5 wt%. Unfortunately, this work does not contain any data concerning the composition of the additives used; it is only noted that all additives contain EVA copolymers. The influence of EVA copolymer on CFP of the blends of waste cooking oil BD (~42% of saturated methyl esters, CP = 5 °C, PP = 2 °C) with petroleum diesel (CP = −4 °C, PP = −8 °C) was studied by Cat et al. [103]. They found that EVA copolymer, which is effective PPD for petroleum diesel (reducing of CP, CFPP and PP by 8, 11 and 10 °C, respectively, when 0.08 wt% of the additive was used), maintains its efficiency for BD/petroleum diesel blends that contain 40 vol% of BD and less. For B20 blend (20 vol% BD) and 0.08 wt% concentration of the EVA copolymer, the lowering of CP, CFPP and PP was the same 8, 11 and 10 °C; for B40 blend, the effect was significantly weaker (6, 6 and 5 °C, respectively).

Note that the impact of the EVA copolymers on CFP of the blends of rapeseed oil methyl esters (CP = −4 °C, CFPP = −11 °C) with petroleum diesel (CP = −3 °C, CFPP = −5 °C) was demonstrated by Davies et al. back in 1998 [155]. For pure rapeseed oil BD, the lowering of CFPP was only 2 °C (320 ppm loading of the EVA copolymer, 36 wt% vinyl acetate, *M*_n_ = 2.4 kDa), while for B50 blend, CFPP was reduced from −10 °C to −27 °C.

The addition of 0.03 wt% poly(methyl acrylate) reduced the PP, CP, and CFPP of coconut BD (90.2% saturated, 7.7% monounsaturated and 2.1% polyunsaturated FAMEs) blend with petroleum diesel (20 vol% of BD) by 9 °C, 3 °C and 8 °C, respectively [156]. Very close results were reported for the same blend in [157].

Cold flow properties of BD blends with petroleum diesel in the presence of poly(methyl acrylate) additive were studied by Islam et al. [158]. *Cocos nucifera* (coconut) BD (89.2 wt% of saturated FAMEs, CP = 14 °C, PP = 13 °C) and *Calophyllum inophyllum* BD (32.9% of saturated FAMEs, CP = 1 °C, PP = −3 °C) were mixed with petroleum diesel (CP = −5 °C, PP = −5 °C), the maximum effect of the additive in B10–B30 blends was detected for ~0.03 wt% concentrations. The best results were obtained for B20 blends; the CP, PP and CFPP were reduced by 6 °C, 9 °C and 12 °C, respectively, for coconut BD blend and by 5 °C, 6 °C and 6 °C, respectively, for *Calophyllum inophyllum* BD blend.

Palm oil BD blends with petroleum diesel in the presence of a series of low-MW poly(alkyl acrylates) and poly(alkyl methacrylates) demonstrated a significant reduction in PP. The best results were obtained for the copolymer of poly(acrylic acid) and poly(tetradecyl methacrylate) used in 0.1 wt% concentration: for the B5 blend, the reduction was 26 °C, for the B20 blend, it was 7 °C [107]. Terpolymers of C12–C15 alkyl methacrylates, C18–C18 alkyl methacrylates and methyl methacrylate (75:15:10 by weight, *M*_n_ = 56 kDa, *Ð*_M_ = 2.3) reduced PP of palm oil-based B20 blend by 3 °C [159].

**Scheme 8:**
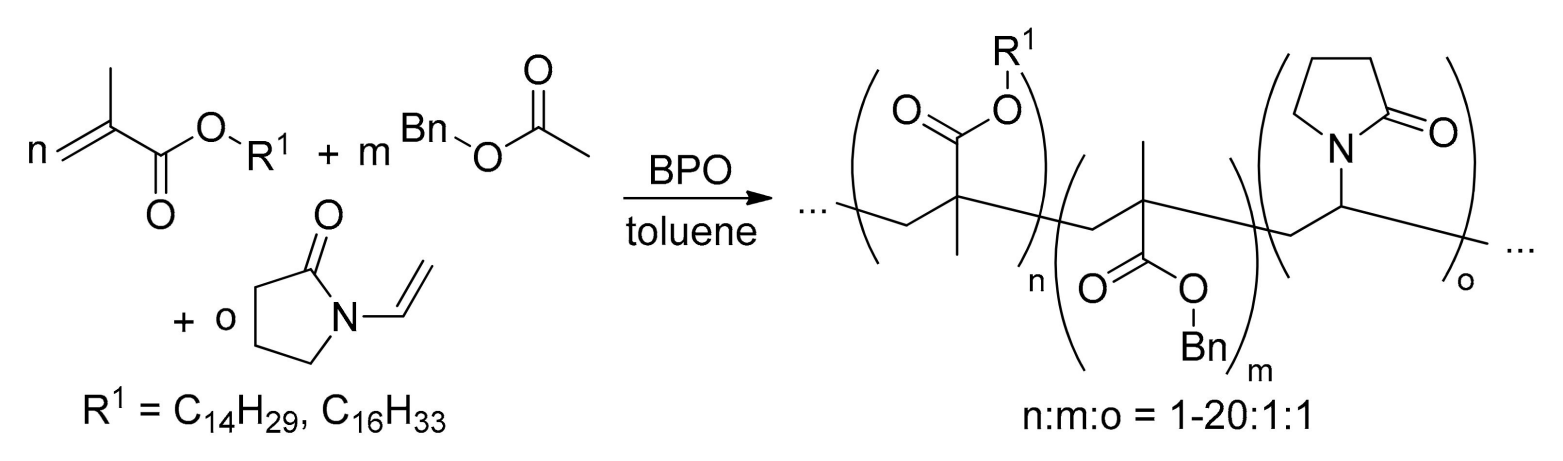
The synthesis of methacrylate/*N*-vinylpyrrolidone terpolymers [160].

Lin et al. recently reported the synthesis of the terpolymers of C14 or C16 *n*-alkyl methacrylate, benzyl methacrylate (BM) and *N*-vinylpyrrolidone (NVP) [160] by free-radical copolymerization on the basis of 1–20:1:1 comonomer ratios (Scheme 8). Similar copolymers, based on C12, C14, C16 and C18 alkyl methacrylates, were studied by Su et al. as PPDs for the blends of the waste cooking oil BD (38.4 wt% of saturated FAMEs, CP = 7 °C, PP = 5 °C) with petroleum diesel (CP = −1 °C, PP = −12 °C) [161].

**Figure 12:**
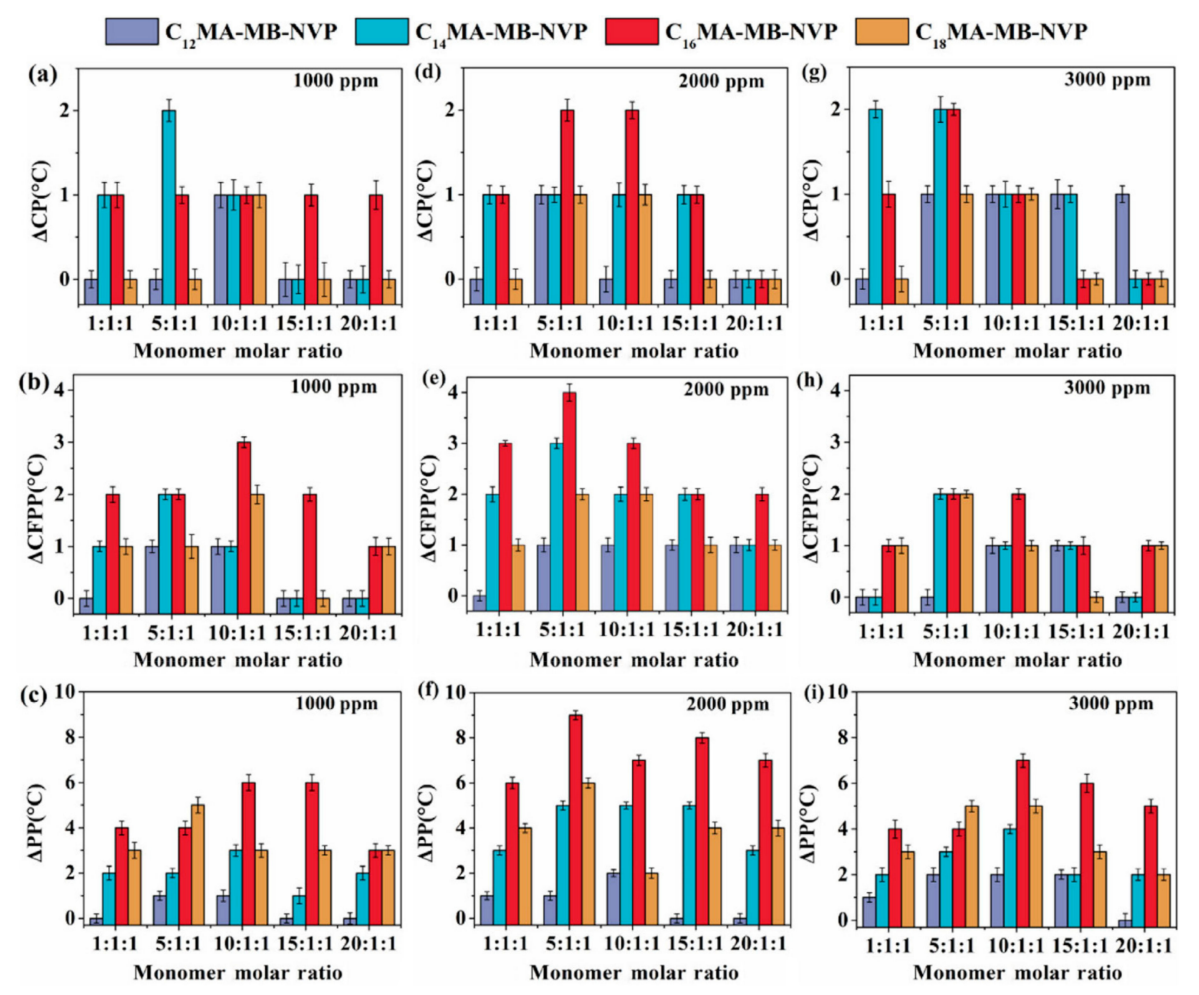
Effects of 1000 ppm (**a**–**c**), 2000 ppm (**d**–**f**), and 3000 ppm (**g**–**i**) RMC-BM-NVP terpolymers on ΔCP, ΔCFPP, and ΔPP of B20 (RMCMB-NVP = methacrylate-benzyl methacrylate-*N*-vinyl-2-pyrrolidone terpolymers, R = C12, C14, C16, C18). Reprinted with permission from [161]. Copyright (2020) Wiley-VCH Verlag GmbH & Co.

The results of this study for B20 blends are presented in Figure 12. When the terpolymers have different monomer proportions, with decreasing ratio of the NVP comonomer, the depressing effect initially increased and then decreased. When the *n*-alkyl methacrylate/BM/NVP molar ratio of the terpolymer was 5:1:1, ΔCFPP had the best reducing effect. The best results were achieved when C16MA-BM-NVP copolymers were used at the dosage of 2000 ppm (0.2 wt%). This result was attributed by the authors to the principle of carbon chain matching: the long carbon chain of C16MC-MB-NVP co-crystallizes with high melting point methyl palmitate, and the polar group of the PPD increases the dispersion of wax crystals. The further improvement of the CFP of B20 blends was achieved through the use of dispersants such as fatty alcohol polyoxyethylene ethers.

## 6. Conclusions and Perspectives

The cold-flow properties of biodiesel have an important role in determining its suitability as alternative fuel or fuel component in moderate temperature climates. The use of polymer additives, pour point depressants and dispersants can often provide a substantial improvement of the CFP of BD. It is possible that polymeric cold-flow improvers can enhance the other characteristics of BD and BD/petroleum diesel blends; for example, the combined effect of hydroquinone-functionalized poly(acrylate)s as CFIs and antioxidants were demonstrated very recently [137].

When one compares the performance of polymer CFIs in BD and in petroleum diesel, the fact is many types of additives are highly effective in petroleum diesel and demonstrate little impact on the CFP of BD. However, these additives are fully effective if used in BD/petroleum diesel blends containing no more than 20 vol% of BD. Perhaps, it is exactly blending with petroleum diesel in combination with the use of polymer CFIs that would increase the global use of BD [30].

We also note that the experimental data, presented in several articles, devoted to the application of the polymer additives for the improvement of the CFP of BD [32,97,98,99,102,108,134,151,156,158,162,163,164], are scientifically irrelevant in the context of our review due to lack of the objective characteristics of the polymers under study: the references on the trade names of the commercial additives are clearly insufficient, given in mind that the recipes of such additives are commercial secrets. Undoubtedly, the high relevance of the biodiesel thematics (Figure 3) attracts the researchers by the possibility of the publication of raw and incomplete results as fully fledged research papers, and in some cases, the patent literature contained more comprehensive data (Table 3). It was to be hoped that further contributions in the field of the improvement of BD characteristics would be made at a more senior level that had long been accepted in top-rated studies of petrochemical products.

We are assuming that the further development of the polymer cold-flow improvers for biodiesel and biodiesel blends should include a more detailed and precise analysis and discussion of the nature of additive, combined with modern research methods (precision rheometry, DSC, SFC NMR, etc.). The global objective is to create the library of polymer cold-flow improvers for petroleum diesel, biodiesel and petroleum diesel /biodiesel blends, which would ensure economically viable and technically competent employment of biodiesel in the global fuel industry.

## Figures and Tables

**Table 1:** FA composition of different oils [16].

Acid Name		Percent Composition in Oil ^1^												
Common	Lipid	RS	CA	CR	SU	SB	P	PK	OL	PN	CO	SA	TL	AlC ^2^
Caproic	6:0	–	–	–	–	–	–	tr–1.5	–	–	0–0.8	–	–	–
Caprylic	8:0	–	–	–	–	–	tr	3–5	–	–	5–9	–	–	–
Capric	10:0	–	–	–	–	tr	tr	3–7	–	–	6–10	–	–	–
Lauric	12:0	–	–	–	–	tr	tr	40–52	–	–	44–52	–	tr–0.2	–
Myristic	14:0	–	–	tr–1.7	–	tr	0.5–6	14–18	0.1–1.2	tr–1	13–19	tr	2–8	2.0
Palmitic	16:0	1–3	4	8–12	3–6	7–11	32–45	7–9	7–16	6–9	8–11	3–6	24–37	19.6
Stearic	18:0	0.4–3.5	2	2–5	1–3	2–6	2–7	1–3	1–3	3–6	1–3	1–4	14–29	3.3
Arachidic	20:0	0.5–2.4	–	tr	0.6–4	0.3–3	tr	tr–1	0.1–0.3	2–4	0–0.4	tr–0.2	tr–1.2	–
Behenic	22:0	0.6–2.1	–	tr	tr–0.8	tr	–	–	–	1–3	–	–	–	–
Palmitoleic	16:1	0.2–3	–	0.2–1.6	tr	tr	0.8–1.8	tr–1	tr	tr–1.7	0–1	–	1.9–2.7	0.8
Oleic	18:1	12–24	61	19–49	14–43	15–33	38–52	11–19	65–85	53–71	5–8	13–21	40–50	5.7
Gadoleic	20:1	4–12	–	–	–	–	–	–	–	–	–	–	–	0.1
Erucic	22:1	40–55	tr	–	–	–	–	–	–	–	–	–	–	–
Linoleic	18:2	12–16	21	34–62	44–75	43–56	5–11	0.5–2	4–15	13–27	tr–2.5	73–79	1–5	11.8
Linolenic	18:3	7–10	9–12	tr	tr	5–11	tr	–	tr–1	tr	–	tr	–	22.3
Timnodonic	20:5	–	–	–	–	–	–	–	–	–	–	–	–	1.3

^1^ Oils: RS—rapeseed, CA—canola, CR—corn, SU—sunflower, SB—soybean, P—palm, PK—palm kernel, OL—olive, PN—peanut, CO—coconut, SA—safflower, TL—tallow and AlC—algae Chlorella. ^2^ Contains 3.6% 16:2 and 12.0% 16:3 acids.

**Table 2:** Melting points of FAMEs [15,89].

	M. P. (°C)			M. P. (°C)	
Saturated Esters	Me	Et	Unsaturated Esters	Me	Et
8:0	−37.4	–44.7	16:1 Δ9c	−34.1	–36.7
10:0	−13.5	–20.4	18:1 Δ6c	−1.0	–7.7
12:0	4.3	–1.8	18:1 Δ9c	–20.2	–20.3
14:0	18.1	12.5	18:1 Δ9t	9.9	4.2
16:0	28.5	23.2	18:1 Δ11c	−24.3	–36.5
18:0	37.7	32.9	18:2 Δ9c, Δ12c	−43.1	–56.7
20:0	46.4	41.3	18:3 Δ9c, Δ12c, Δ15c	–52	–61.7
22:0	53.2	48.6	20:1 Δ11c	−7.8	–8.8
24:0	58.6	55.9	22:1 Δ13c	−3.1	–10.5

**Table 3:** The summary of the data on polymer CFIs for biodiesel.

Biodiesel		Polymer CFIs Added			ΔCP, °C	ΔPP, °C	ΔCFPP, °C	Reference	
Name	Saturated FAMEs wt%	Type	Composition/ MW/*Ð*_M_	Concentration, wt%				No.	Rating ^1^
Canola	6.2	EVA	– ^2^/–/–	1	0	11	–	100	Q1
Canola	6.2	poly(acrylate)	+/+/+	0.5–1	–	30	–	100	Q1
Canola	–	poly(acrylate)	–/–/–	2	5	–	–	135	Q1
Canola	–	MA/C_18_OCH=CH_2_	+/+/–	1	–	3	–	100	Q1
Rapeseed	–	MA/olefin/acrylate	–/–/–	0.5	–	2	4	151	Q3
Rapeseed	~10	poly(acrylate)	+/+/+	0.3–0.5	–	9	6	126	P
Rapeseed/soybean	10.0	EVA-g-acrylate	+/+/+	0.5	–	15	5	126	P
Rapeseed/soybean	10.0	EVA-acrylate	+/+/+	0.3	2	–	9	126	P
Soybean	14.3	EVA	–/–/–	0.01	–	2	1	106	Q1
Soybean	17.4	poly(acrylate)	–/–/–	0–1	–	–	0	133	Q1
Soybean	–	poly(acrylate)	–/–/–	0.5–1	–	12	–	110	P
Soybean	–	poly(acrylate)	+/+/+	0.1	–	5	–	136	P
Soybean	–	poly(acrylate)	+/+/+	0.2	3	9	–	137	Q1
Soybean	14.5	poly(acrylate)	–/–/–	0.5	–	30	–	24	Q1
Palm	43.6	polyolefin	1-decene/–/–	0–2	0	0	0	105	Q1
Palm	46	EVA	–/–/–	1	–		6	127	Q1
Palm	–	poly(acrylate)	+/+/+	0.01–0.1	–	0	–	107	Q3
Palm	–	poly(acrylate)	–/–/–	0.5–1	–	9	–	110	P
Palm	36.9	poly(acrylate)	+/–/–	0–2	0	0	0	105	Q1
Palm	36.9	MA/C_18_	+/–/–	2	–	6	–	105	Q1
Tobacco seed	12	MA/C_18_	+/–/–	1	–	–	7	149	Q1
Waste cooking	–	polyolefin	–/–/–	0.1	–	2–3	2–3	108	Q2
Waste cooking	–	polyolefin	–/–/–	0.02–0.08	1	1	3	98	Q1
Waste cooking	–	EVA	–/–/–	0.02–0.08	2	6	2	98	Q1
Waste cooking	43.5	EVA	–/–/–	0.02–0.08	0	3	1	103	Q1
Waste cooking	–	poly(acrylate)	–/–/–	0.02–0.08	0	8	6	98	Q1
Waste cooking	32	poly(acrylate)	–/–/–	0.5	–	–	7	134	Q3
Waste cooking	–	MA/acrylate/C_14_N	+/–/–	0.05	–	13	–	104	Q3
–	–	MA/C_18_/ON ^3^	+/+/+	0.01	5	–	–	150	P
–	–	acrylate/acrylamide	–/–/–	0.5	–	<5	<5	109	P

^1^ Q1–Q4—SJR rating, https://www.scimagojr.com/journalrank.php, accessed on 1 April 2021. P—patent or patent application. ^2^ No data presented. ^3^ Modified by aliphatic alcohols and amines.
